# Electrified Solar Zero Liquid Discharge: Exploring the Potential of PV-ZLD in the US

**DOI:** 10.1021/acs.est.4c00494

**Published:** 2024-05-03

**Authors:** Rodrigo A. Caceres Gonzalez, Marta C. Hatzell

**Affiliations:** ‡ George W. Woodruff School of Mechanical Engineering, Georgia Institute of Technology, Atlanta, Georgia 30332, United States; † School of Industrial Engineering, Faculty of Engineering and Science, Universidad Diego Portales, Santiago 8370191, Chile; § School of Chemical and Biomolecular Engineering, GeorgiaInstitute of Technology, Atlanta, Georgia 30332, United States

**Keywords:** Solar desalination, Zero liquid discharge, Multiobjective optimization, Brine Management, Water-Energy nexus, Decarbonization, Decentralized brine management

## Abstract

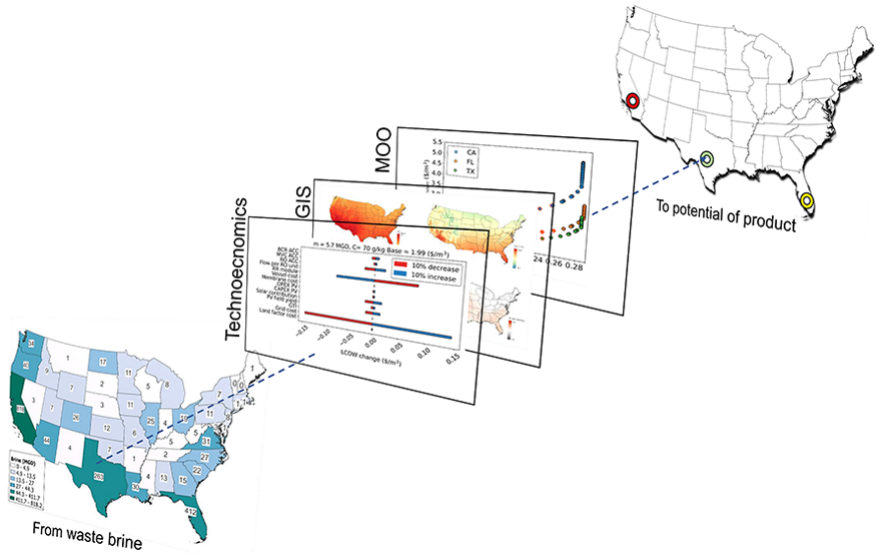

Current brine management strategies are based on the disposal of brine in nearby aquifers, representing a loss in potential water and mineral resources. Zero liquid discharge (ZLD) is a possible strategy to reduce brine rejection while increasing the resource recovery from desalination plants. However, ZLD substantially increases the energy consumption and carbon footprint of a desalination plant. The predominant strategy to reduce the energy consumption and carbon footprint of ZLD is through the use of a hybrid desalination technology that integrates renewable energy. Here, we built a computational thermodynamic model of the most mature electrified hybrid technology for ZLD powered by photovoltaic (PV). We examine the potential size and cost of ZLD plants in the US. This work explores the variables (geospatial and design) that most influence the levelized cost of water and the second law efficiency. There is a negative correlation between minimizing the LCOW and maximizing the second-law. And maximizing the second-law, the states that more brine produces, Texas is the location where the studied system achieves the lowest LCOW and high second-law efficiency, while California is the state where the studied system is less favorable. A multiobjective optimization study assesses the impact of considering a carbon tax in the cost of produced water and determines the best potential size for the studied plant.

## Abstract 2

We evaluate the potential of PV-ZLD to treat desalination brine produced in the US. The feasibility of PV-ZLD is found to depend significantly on the cost of energy received from the grid and performance of the preconcentration RO stage. Large-scale PV-ZLD systems have the potential to recover more than 1 billion gallons of freshwater per day in the US, based on current brine production in three states. Implementing a carbon tax increases the solar contribution in the design optimization

## Introduction

Every day, the desalination industry in the contiguous US rejects about 2 billion gallons of brine (or 7.8 million cubic meters). California, Florida, and Texas are responsible for rejecting more than 70% of the total desalination brine in the contiguous United States. The approximate percentage of brine produced in each state is 40%, 23%, 13%.^1^ For plants close to the coast, the direct discharge of brine to the sea is the most commonly used brine management strategy. For the inland areas, the predominant disposal strategy is deep-well injection.^2,3^ Both management strategies have negative social and environmental impacts.^4−6^ Without consolidated brine management, there are also logistical challenges. The disposal of water transported away from a desalination site is an energy-intensive and counterproductive process that loses valuable water. The use of desalination brine as an anthropogenic stream with high salinity in the production of power from a salinity gradient is an alternative for brine management. However, new technologies are needed to prevent membrane contamination and reduce costs to achieve large-scale applicability.^7,8^ The study of synergies between the mixture of effluent streams has been studied in the literature with the combination of SWRO brines diluted with wastewater. This might lead to reductions in the system’s specific energy consumption.^9^

Zero liquid discharge (ZLD) has recently gained interest as a strategy to completely recover water from desalination brine.^10^ The primary challenge in developing a ZLD industry lies in the substantial energy consumption associated with existing technologies, which escalates with the concentration of brine treated.^10,11^ The expansion of the treatment of high-salinity brine through desalination or reuse requires improvement of materials and processes with the aim of reducing capital and operating costs.^12^ New technologies have emerged to improve this, such as forward osmosis, solvent extraction, and new evaporative methods, that use renewable energy. However, none had large-scale commercial applications.^13−19^

From a thermodynamic point-of-view, the minimum energy required to separate all salts from brine in the United States is equal to 7.8 TWh (Figure S1). This is equivalent to 0.8% of the total electricity (consumption) in the industrial sector in the United States during 2021, according to the Energy Information Administration (EIA).^20^ Since the thermodynamic minimum is unrealistic to attain, the actual minimum energy requirement is closer to double this value. In ZLD, the process with the greatest potential for improvement is the brine concentration.^21^ Here, the brine reaches a saturation point in terms of solubility.

Without considering a technological breakthrough for saturating the brine in one step, the best strategy for decreasing the energy requirements of ZLD is the hybridization of different technologies based on operation salinity limits. Hybridization reduces the total specific energy consumption by combining the most efficient technologies for a particular feeding salinity in series.^22,23^ There are numerous alternative hybrid methods based on membranes to concentrate brine, but none are in the commercial state. The use of osmotically assisted reverse osmosis (OARO) and low salt rejection reverse osmosis (LSRRO) systems can decrease the specific energy consumption by 50% compared to traditional brine concentration systems.^24,25^ However, membrane-based methods for the treatment of high-salinity brine had particular challenges related to fouling, which restricts water flux.^26^

Combining freeze desalination, membrane distillation, and crystallization can reach ZLD levels and use renewable energy as an energy source. However, energy consumption is greater than RO-based systems.^27^ The use of electrodialysis, combined with traditional membrane processes, has shown a potential to concentrate brine with lower energy requirements than conventional systems.^28,29^ Today, the most mature technologies for ZLD are electricity-driven, combining reverse osmosis with mechanical vapor compressors and crystallizers.

As more brine with high salinity is treated, the system will require more energy, putting additional stress on the grid.^10^ Here, the integration of ZLD with solar energy is a strategy that may alleviate dependence on the current grid. However, establishing the ideal solar field size relies on the anticipated energy consumption of the ZLD plant, which is in turn influenced by the flow and concentration of brine. Second-law efficiency serves as a crucial benchmark tool for comparing desalination technologies with different primary energy sources.^30^ This metrics allows to compare electricity driven technologies with heat driven technologies fairly.^31^ However, second-law efficiency should not be evaluated standalone and the focus should also be in reducing the cost.^32^ In this work, we hypothesize the existence of a correlation when comparing the Levelized Cost of Water (LCOW) and second-law efficiency for a technologically mature ZLD system driven by solar energy, where currently there is a gap in identifying potential correlations between the cost of produced water and the thermodynamic performance.

This work evaluates the potential of solar driven hybridized desalination as a sustainable application in the contiguous United States to treat brine rather than reject it. The potential of a solar-driven ZLD must be analyzed by combining different performance metrics and parameters, which are usually location-dependent. This work studies the dependence of the second-law efficiency and levelized cost of water for the production of freshwater from brine. For this, geospatial information must be integrated into the analysis. The consideration of multiple criteria allows us to avoid being misled in decision making.^33^

We investigated the sensitivity of design variables related to the Zero Liquid Discharge (ZLD) system and geospatial information specific to the studied state. A multiobjective optimization framework determines the potential size, taking into account both location and operational variables. The aim is to maximize the thermodynamic performance of the ZLD system while minimizing the cost of water produced when an environmental tax. These results serve as a threshold for implementing a spatially constrained clustering algorithm to determine the required number of plants in the studied regions. The studied system uses high-pressure reverse osmosis (HPRO) as the preconcentration. While Conventional RO has a salinity limit of 70 g/kg,^10,34^ we assumed HPRO as a system that can operate with pressures up to 120 bar.^34^

## Methodology

This work evaluates an electrified hybridized system for zero liquid discharge (Figure S2). The system has three stages operating in series. A two-stage RO system treats the inlet brine increasing the concentration of the brine before entering an MVC brine concentrator which produces nearly saturated brine before entering a crystallizer. The system produces freshwater and solid salts as outputs. The energy is supplied by a photovoltaic system that uses the grid as a backup. In the United States, connecting devices directly to the electricity grid has been shown to be a competitive alternative to using fully renewable systems that rely on battery storage, due to the current cost of battery technologies.^35^ The development of a computational model aims to estimate pertinent parameters and metrics for designing and installing a system in a specific location.

### Computational Model of the Electrified Zero Liquid Discharge System

During the preconcentration stage (passing through the RO system), the effluent brine is the feedwater entering the intake pump. The first pressure exchanger (PX1) mixes 60% of the feedwater with the remaining feedwater before entering the first RO module (RM1). The second pressure exchanger (PX2) mixes 60% of the brine of the first RO module with the remaining brine before entering the second RO module (RM2). In the RO module, the flow is evenly distributed into multiple units consisting of 43 pressure vessels, each with seven membrane elements. The brine from the second RO module flows into the pressure exchangers as the high-pressure fluid, and the regenerators (R1 and R1) as low-temperature fluid. Each unit treats 2000 m^3^/h (12.7 MGD). In the concentration step, the brine enters the evaporator. Here, the vapor from the compressor transfers heat to the evaporation process. The condensed vapor mixes with the permeate of the RO modules, producing freshwater. Nearly saturated brine (260 g/kg of concentration) leaves the evaporator and condenser and is transferred to the crystallizer. A heater uses the vapor produced in the same crystallizer to increase the temperature of the saturated brine before it reentering. In this device, part of the brine evaporates, producing a high-concentration slurry, where crystal salts are formed. The vapor, compressed into the heater, condenses and produces freshwater. Crystals separate from the slurry in the separator, producing solid salts. The remaining slurry flows through the recirculation pump to mix with the incoming brine to repeat the cycle. The power requirements in this system are energy consumption from pumping and compressing in the concentration and crystallization steps. A photovoltaic system without battery storage but grid energy backup supplies the required power to the system. The size of the photovoltaic is a variable in this work ranging from 0 to 1, where 0 indicates a full grid system and 1 indicates a full PV driven system.

The computational model, developed in the Engineering Equation Solver software,^36^ solves for energy and mass balances providing second law efficiency, power consumption, freshwater, and salt produced as a function of input brine flow (m^3^/h), and concentration (g/kg). Correlations and data from literature regarding thermo-physical and thermodynamics properties of brine complement EES in-built functions for estimating the required parameters.^37−43^ Furthermore, the correlations available in the literature provide the capital cost for every studied system (see Supporting Information methodology section). The relevant economic metrics obtained are the annualized capital cost for the preconcentration (RO), concentration (MVC), and crystallization (BCr) subsystems. In this study, due to the difficulty of defining the composition of every brine produced in the US, based on feedwater and location, we assumed an aqueous sodium chloride mixture. This is due to the availability of thermophysical properties correlations and data that allows for a standardization in the analysis.^37−43^ While this affects the separation energy, depending on the level of concentration of every component, this impact may not be significant. For instance, for well brine production (associated with shale gas production), the mixtures can be considered a Ca–Na–CL, with a low impact of Ca concentration levels in the properties of the mixture.^44^ Then, an aqueous sodium chloride solution is selected. Regarding brackish water thermophysical properties (or feedwater with concentration lower than 35 g/kg, literature suggest that from for typical brackish water compositions, feed streams could be approximated as sodium chloride solutions.^45,46^ Sodium and/or chloride are commonly the dominant species in brackish water even with the increase’s presence of calcium and sulfates.^47,48^ The variable recovery ratio based on technology and feedwater allows to estimate the power consumption (see Supporting Information methods and Table S1).^49^ The performance difference in desalination technologies when treating different feed streams in this work is based on the total dissolved salts (TDS) and the recovery ratio. Under this context, the brine is considered nearly saturated at 260 g/kg.^50^

### Metrics of Study

The developed model in EES estimates the second-law efficiency (η_II,ZLD_) and levelized cost of water (LCOW).

The second law efficiency is a function of the specific energy consumption of the system and the minimum required energy for separating the salts from the water as follows
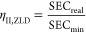
1where SEC_real_ is the ratio between the power consumption and the freshwater produced, and SEC_min_ is the minimum energy required as a function of the Gibbs free energy of the inlets and outflows.^51,52^ For brine concentration systems, SEC_min_ estimations considers a finite recovery (*rr* > 0).^30^
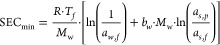
2where *R* is the universal gas constant, *T*_*f*_ the feed temperature, *M*_*w*_ is the molar mass of water, *b*_*w*_ the molality of the inlet (feed) brine, *a*_*w*,*f*_ the activity of the inlet brine, *a*_*s*,*p*_ the activity of the salt in a saturated solution, and *a*_*s*,*f*_ the activity of the salt in the feed brine.

The levelized cost of water considers the size of the PV field (which is a direct function of the power consumption), the total freshwater production, and the annualized capital cost as follows
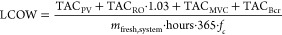
3where TAC_PV_, TAC_RO_, TAC_MVC_, and TAC_BCr_ are the total annualized cost of the PV field, RO, MVC, and BCr subsystems. The factor of 1.03 represents a conservative difference between a traditional RO system and one working as high-pressure reverse osmosis (HPRO) where the upper pressure increases to 120 bar.^50^*f*_*c*_ is the capacity factor of the system assumed as 0.9. The total annualized cost is the summation of the annual operational cost (TOC) and the annualized capital cost as follows
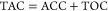
4
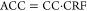
5where CRF is the capital recovery factor used to annualizing the total capital cost of the system, which is equal to the sum of the purchase cost of each device. The annualized capital cost per device are corrected to the year 2021 using the CEPCI correction factor (see Supporting Information methodology section)^53,54^
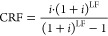
6where *i* is the interest rate assumed as 0.05 and LF the lifetime of the plant, assumed as 20 years. The CAPEX and OPEX for the solar field are 1549 $/kWp and 14 $/kWp/year.^55,56^ The total annualized cost for the PV field also considers the land annualized cost (see Supporting Information methods section).

As mentioned, second-law efficiency holds significant value due to its independence from economic parameters.^30^ Consequently, this work explores the inherent correlation between this thermodynamic performance metric and the leveled cost of water.

### Model Inputs

The developed model takes as input design operational and geospatial parameters. The goal is to evaluate the influence of both, system design and location on the assessment of ZLD potential in the US. The selected parameters for study, along with their base values are summarized in Table 1 and categorized by system (for design and operational input parameters) and geospatial (for location related parameters) inputs. These values are based on literature standard (for design and operational parameters in the RO, MVC and Brine crystallizer subsystems) and the US average for geospatial variables (Figure S3a, S3b, S3c, and S3d^20,57,58^).

**Table 1:** Model Inputs for System and Geospatial Variables^a^

Parameter	Category	Base value	Unit
Solar contribution	System	0.5	-
Land cost	Geospatial	8.0	ln($/ha)
Electricity grid cost	Geospatial	7.715	cents/kWh
PV yield	Geospatial	4.35	kWh/kWp
GTI	Geospatial	1869	kW/*m*^2^
CAPEX solar	System	1549	$/kWh
OPEX solar	System	14	$/kWh
Membrane cost	System	1200	$
Pressure vessel cost	System	1000	$
RR module	System	0.3	-
Flow per unit	System	12.7	*m*^3^/*h*

a This set of values represents the base case of the studied system.

### US Analysis and Database

For the analysis in the US, the brine produced is calculated as a function of the reported capacity of the desalination plants and the average recovery ratio of desalination technologies based in inlet salinity^49^ as follows
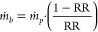
7where  is the brine produced,  is the desalination plant capacity and RR is the recovery ratio of the technology (Table S1).^49^ The total brine produced by each state is the sum of the individual brine volumes generated by all of the reported desalination plants within the contiguous US that are located in each respective state.

The desalination plants database is provided by Global Water intelligence.^1^ This database contains information about the plant status, capacity, location (represented in latitude and longitude, country, and region), main desalination technology, customer type (the use of the produced water), and feedwater used in each desalination plant. This database is used in literature as information source of the desalination industry.^33,34,49,59^ We filtered this database for plants that are reported as online, presumed online, in construction, and planned, obtaining a total of 13567 plants, then choosing only the 1774 plants reported in the US.

### Multiobjective Analysis

With the majority of brine production occurring in three states (76%), and considering the existence of a correlation between performance and cost (second-law efficiency and LCOW), this section assesses the potential of the studied system in these states while varying one of these metrics.

A Pareto front study allows us to evaluate the system’s potential in different states by varying the brine flow to be treated (ranging from 0.6 to 38 MGD), brine concentration (ranging from 25 to 120 g/kg), PV yield (from the minimum to the maximum for each state), and solar contribution (ranging from 0.1 to 0.95). The Pareto front for each state takes into account location variables such as energy grid cost, Global Horizontal Irradiance (GTI), ambient temperature, and land cost and 10000 simulations per state. Our analysis uses a greedy heuristic, based on the minimization of a weighted objective function
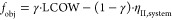
8where γ is a priority parameter varying form 0 to 1. When γ = 0, the problem aims to identify the system configuration that maximizes the second-law efficiency. When γ = 1, the problem aims to identifying the system configuration that minimizes the LCOW. As we aim to evaluate the potential best design of the studied system as a sustainable alternative to brine management methods in these specific states, we include in this section a carbon tax for penalizing the LCOW as follows
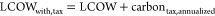
9
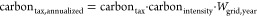
10where carbon_tax_ is the carbon price assume as 0.1372 $ per kg of CO_2_,^60^ carbon_intensity_ is the grid carbon intensity, which varies per state,^61−63^ and *W*_grid,year_ is the total power required from the grid by the system. This value is the difference between the power requirements of the system and the power contributed by the solar field. The rationale behind this tax is to identify the best configuration, per state, that increases the contribution of the PV field and decreases the grid dependence.

## Results

**Figure 1:**
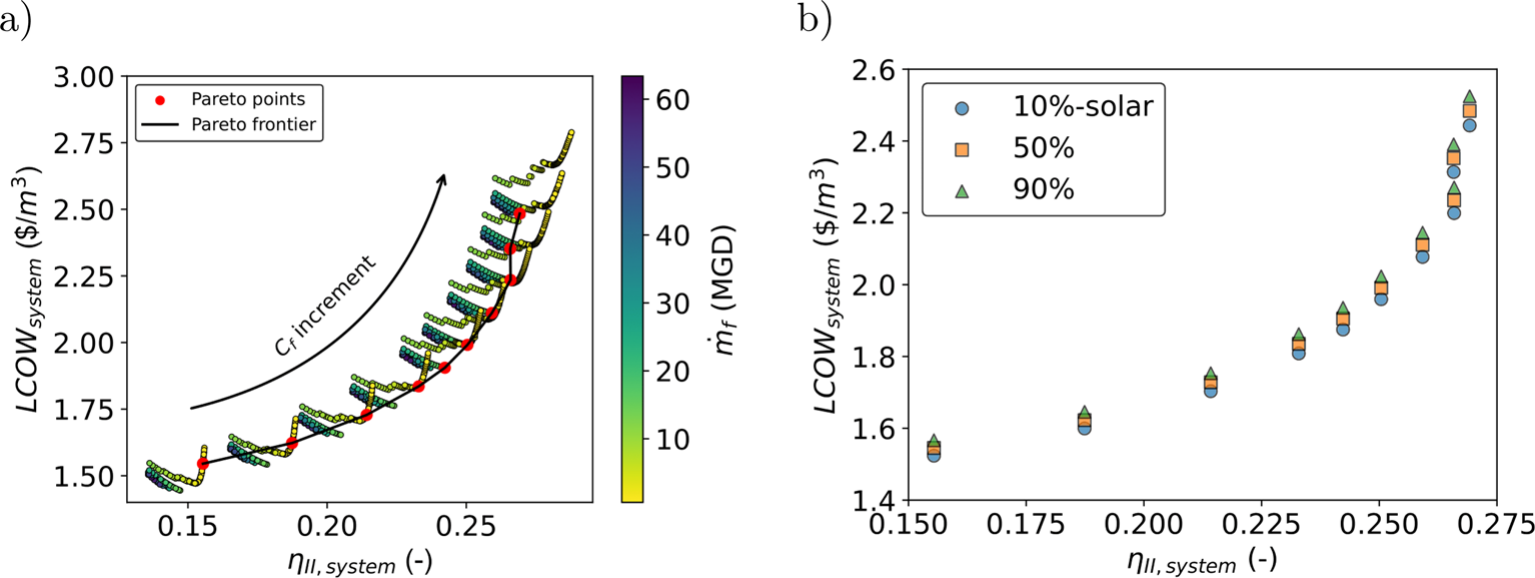
LCOW and second-law efficiency as a function of feed flow and concentration for a PV field contributing to (a) 50% of the system power requirements. The red points and black line represent the Pareto points and front. The color bar follows the increment in feed flow. Panel b shows the Pareto Front of the system studied with a solar contribution of 10%, 50%, and 90%.

For the system under the base scenario (Table 1), both the brine concentration and feed flow rate significantly influence the metrics studied in the computational model across different solar contributions. Varying the inlet brine flow from 0 to 10,000 m3/*h* (0–63 MGD) and concentrations from 25 to 120 g/kg reveals an inverse relationship between second-law efficiency and LCOW (Figure 1). The red points in the figure represent, for each concentration studied, the configurations closer to the ideal solution (minimal LCOW and maximum second-law efficiency).

With an increasing concentration, the optimal feed flow, as determined by the Pareto analysis, increases from 0.8 MGD when brine is treated with a concentration of 25 g/kg to 26.2 MGD when brine is treated with a concentration of 120 g/kg. For concentrations below 67 g/kg, the Pareto point results in an LCOW below 2 $/m^3^. This value is comparable to the average cost of rejection for land application (ranging from approximately 0.74 to 1.95, with an average of 1.35 $/m^3^), deep-well injection (ranging from about 0.54 to 2.65, with an average of 1.6 $/m^3^), and lower than that of evaporation ponds (ranging from about 3.28 to 10, with an average of 6.7 $/m^3^). Surface and sewer discharge costs cannot be compared, as their average cost of rejections is 0.18 and 0.5 $/m^3^, respectively.^11,64^

For all concentrations, the maximum second-law efficiency is achieved with a feed flow below 2 MGD, while the minimum LCOW is attained when treating more than 12.7 MGD. This underscores the correlation between the cost and efficiency in treating brine. When a concentration of 120 g/kg (the maximum salinity in this study) is considered, the second-law efficiency of the ZLD system reaches its maximum. However, in all the cases studied (as shown in Figure 1), the LCOW remains above 2.5 $/m^3^.

The influence of brine flow and concentration on the second-law efficiency and LCOW is the main factor that explains the correlation. Second-law efficiency increases with brine flow and concentration for flows lower than 12.7 MGD. The peak increment in this range is due to the increase in the energy consumption required by the RO subsystem as feed flow increases to 12.7 MGD. As mentioned in the previous section, the number of units in every RO stage depends on the amount of feed treated. Each unit treats 12.7 MGD. As the flow increases from 0 to 12.7 MGD, the pressure drop increases, requiring more energy (Figure S4). The effect on the second-law efficiency of adding additional units in the RO step decreases with an increase in the feed flow. For flows larger than 25 MGD the second-law efficiency depends on concentration the most. On the other hand, power consumption increases with feed flow and concentration increase (Figure S4). This is due to the increase in the pumping power for the RO subsystem (for overcoming the pressure drop and difference in osmotic pressure) and the increased power required by the compressors in the MVC and BCr subsystems. As a consequence, the LCOW increases.

For a solar contribution of 50% (Figure 1a), the LCOW under base case conditions ranges from 1.52 to 2.92 $/m^3^. For the solar contribution of 10% (Figure S5a), the LCOW under base case conditions ranges from 1.5 to 2.89 $/m^3^. For the solar contribution of 90% (Figure S5b), the LCOW under base conditions ranges from 1.54 to 2.95 $/m^3^. The increase in solar contribution implies an increase in PV field nominal size and area. An increase in the PV nominal size increases the total CAPEX and OPEX of the solar field, while a large area increases the land cost portion of the solar field. This increment explain the larger LCOW when solar contribution is 90%. However, the variation is about 2% between minimum and maximum solar contribution, indicating that, for the base case scenario (Figure 1b), the CAPEX and OPEX of PV field does not make unfeasible the implementation of renewable driven ZLD. However, this is considering the grid as energy backup to ensure a continuous operation of the plant during the year.

### Sensitivity Analysis

**Figure 2:**
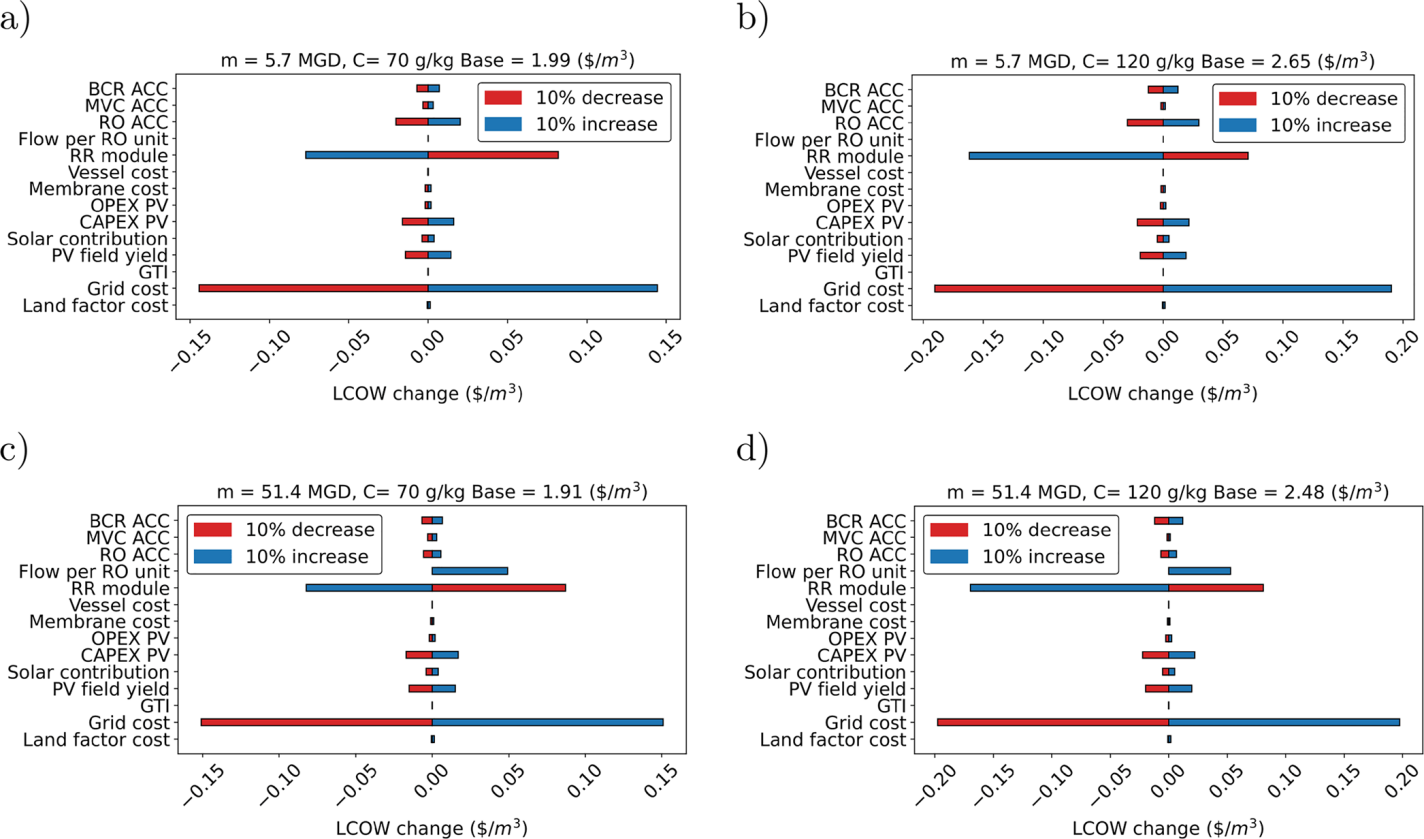
Sensitivity analysis of the ZLD plant with 50% of solar contribution when varying: (a) location related parameters for treating 5.7 MGD at 70 g/kg; (b) location related parameters for treating 5.7 MGD at 120 g/kg; (c) operation and cost parameters for treating 5.7 MGD at 70g/kg; (d) operation and cost parameters for treating 5.7 MGD at 120g/kg. Base case values are presented in Table 1. Each parameter varies 10%.

Intake flow and especially concentration influence the thermodynamic efficiency of the system and the levelized cost. We further explore the potential LCOW and second-law efficiency by a sensitivity analysis around location-design, and operating-cost variables (Figure 2). The location-design variables are the cost of land, the cost of the grid energy, the global tilted irradiation, the PV yield, and the solar contribution. The operating-cost variables are the CAPEX and OPEX for the PV field, the cost of membranes and pressure vessels in the RO units, the recovery ratio of the RO module, the flow per unit in the RO subsystem, and the annualized capital cost (ACC) for the ZLD plant subsystems (RO, MVC, and BCr). The base case conditions represents the average of the contiguous US, which values are summarized in Table 1.

For a ZLD plant, with 50% of solar contribution, treating 5.7 MGD with a concentration of 70 g/kg (seawater brine when recovery ratio is around 50%), the LCOW of the system is 1.99 $/m^3^ (Figure 2a). The grid electricity cost and recovery ratio in the RO subsystem are the most influential parameters from the studied variables. A 10% variation in the cost of electricity from the grid varies the LCOW in ±0.14 $/m^3^ in the same direction. This variable changes the LCOW in 7%. On the other hand, increasing the recovery ratio of the RO subsystem (the step before entering the brine concentrator) decreases the LCOW in 0.077 $/m^3^, while decreasing the recovery ratio increase the LCOW in 0.082 $/m^3^. This variable changes the LCOW by about 4%. Other relevant design and location parameters shown in the figure are the capital cost of the RO and brine crystallizer subsystems, the CAPEX, and the yield of the PV field. However, the influence of this variable changes the LCOW in about 1% from the base value.

For a ZLD plant, with 50% of solar contribution, treating 5.7 MGD with concentration of 120 g/kg (hypersaline brine), the LCOW of the system increases by 33% up to 2.65 $/m^3^ (Figure 2b). The relevant parameters are the same as the previous case with a difference in the recovery ratio of the RO subsystem. A 10% increase in this variable reduces the LCOW in 6%, while a 10% decrease in this variable increases the LCOW in 3%. Treating brine at 120 g/kg in a HPRO system with a 0.33 of recovery ratio, produces brine with a concentration above 260 g/kg (the defined entering concentration to the crystallizer subsystem). Therefore, it can flow directly into the crystallization step without the need of an intermediate brine concentration step. This highlight the importance and potential of HPRO as a preconcentration step and the relevance that that advances in the recovery ratio for HPRO will have allowing to dispose of conventional brine concentrators.

For a ZLD plant, with 50% of solar contribution, treating 51.4 MGD with a concentration of 70 g/kg (seawater brine when recovery ratio is around 50%), the LCOW of the system is 1.91 $/m^3^ (Figure 2c). The grid electricity cost and recovery ratio in the RO subsystem are the most influential parameters from the studied variables. As increasing the inlet brine flow, the flow per RO unit gains in relevance, as the inlet feed is larger than the base value defined (12.7 MGD). A 10% variation in the cost of electricity from the grid varies the LCOW in ±0.2 $/m^3^ in the same direction. This variable changes the LCOW in 8%. On the other hand, increasing the recovery ratio of the RO subsystem (the step before entering the brine concentrator) decreases the LCOW in 0.087 $/m^3^, while decreasing the recovery ratio increase the LCOW in 0.082 $/m^3^. This variable changes the LCOW in about 4%. A decrease in the flow per RO unit does not alter the LCOW in the system, but a 10% increase will increase the LCOW by 0.05 $/m^3^ (a 2.60%). The remaining variables had a 1% or less influence when varying 10%.

For a ZLD plant, with 50% of solar contribution, treating 51.4 MGD with a concentration of 120 g/kg (hypersaline brine), the LCOW of the system is 2.48 $/m^3^ (Figure 2d). As the previous cases, the grid electricity cost and recovery ratio in the RO subsystem are the most influential parameters from the studied variables. A 10% variation in the cost of electricity from the grid, varies the LCOW in ±0.2 $/m^3^ in the same direction. This variable changes the LCOW in 8%. Increasing the recovery ratio of the RO subsystem decreases the LCOW in 0.17 $/m^3^ (a 6.9%), while decreasing the recovery ratio increase the LCOW in 0.082 $/m^3^ (a 3.3%). A decrease in the flow per RO unit does not alter the LCOW in the system, but a 10% increase will increase the LCOW by 0.1 $/m^3^ (a 2.10%). The remaining variables had a 1% or less influence when varying 10%.

The sensitivity analysis shows that, for an equal concentration, as the capacity of the plant increases, the LCOW decreases thanks to scale economies. The most relevant parameter is the concentration feed, followed by grid cost, RO recovery ratio, CAPEX and yield of PV field, and flow per RO unit. The viability and increase in recovery ratio of membrane systems designed for treating hypersaline brine (above 120 g/kg of concentration) will allow replacement of the traditional brine concentrator based on mechanical vapor compression.

From a second-law efficiency perspective, the only relevant parameters are the brine flow, concentration, and recovery ratio of the RO subsystem. This is expected, as second-law efficiency is not influenced by economic parameters but rather by design considerations and fluid properties (Figure S6).

### US Analysis

**Figure 3:**
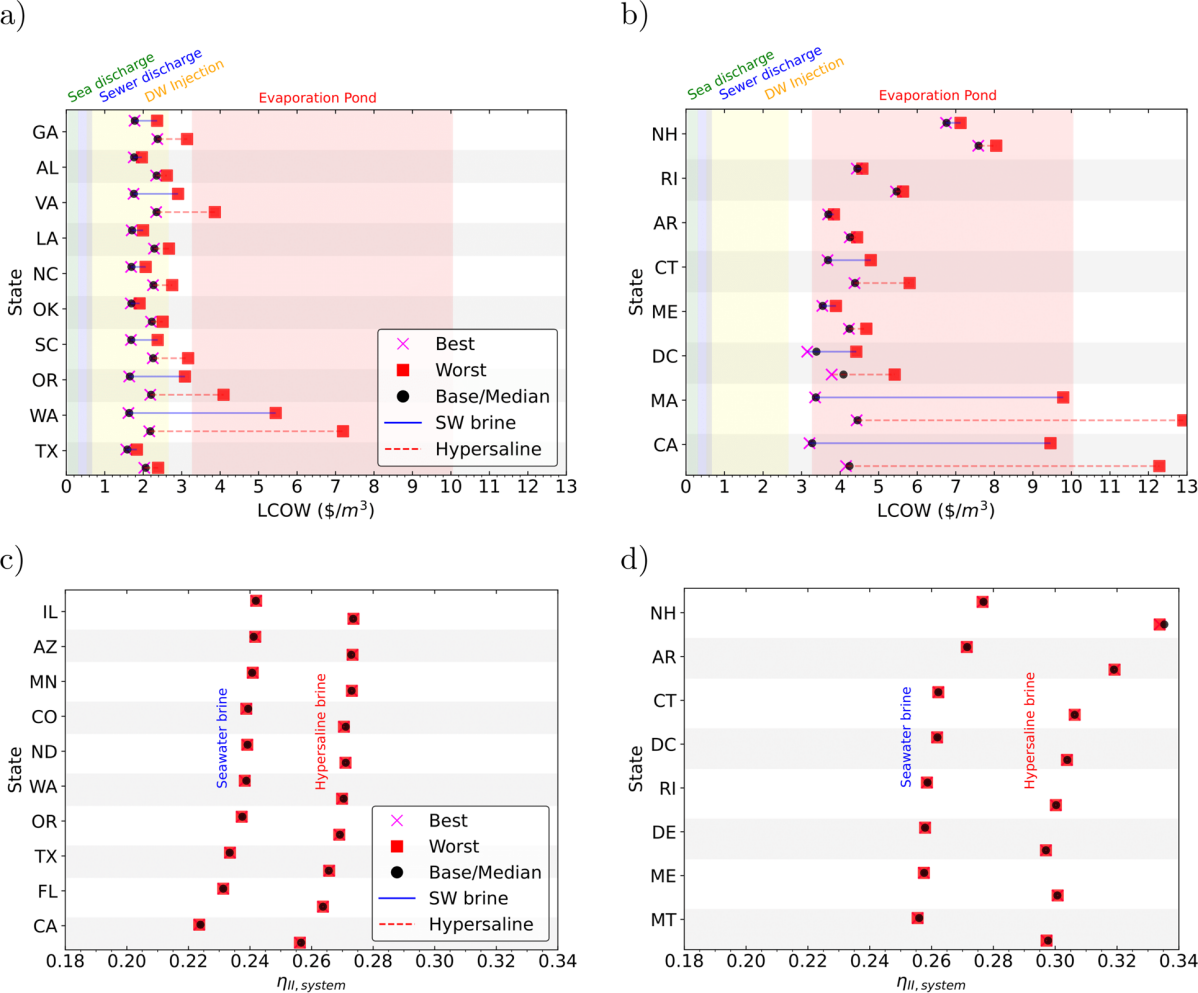
LCOW and second-law efficiency range for the US when considering a plant treating seawater brine (concentration of 70g/kg) and hypersaline brine (concentration of 120 g/kg) with capacity of 10% the produced brine per state. States are ordered from the lowest LCOW and second-law efficiency when considering the median geospatial variables considered (PV yield, GTI, ambient temperature, and land cost) to the highest. Figure also shows worst and best scenario of geospatial variables (i.e, lowest land cost and highest solar potential). (a, b) States with the lowest and higher LCOW and (c, d) the states with the lowest and highest second-law efficiency.

Identified the relevant parameters that influence the two studied metrics in this work, we evaluate the performance of the system, under base conditions, and considering the median values for the geospatial variables in the contiguous United States. Here, the levelized cost of water (LCOW) and second -law efficiency exhibits variation based on geographic location, brine capacity, and the concentration treated (Figure 3). For a system with 50% solar contribution and treating 10% of the total produced brine in each state, with two potential concentrations (seawater brine with 70 g/kg and hypersaline brine with 120 g/kg), Texas emerges as the state with the most economical LCOW, registering at 1.59 $/m^3^ and 2.06 $/m^3^. Following are states: Washington, Oregon, South Carolina, and Oklahoma (Figure 3a). This is attributed to favorable conditions in these specific states, including energy grid pricing and photovoltaic (PV) yield.

Across all of the mentioned states, the baseline LCOW falls within the range of the brine discharge price for deep-well injection and consistently remains below the cost associated with evaporation ponds. This underscores the potential value of brine recovery from the states highlighted in Figure 3a.

Conversely, New Hampshire (NH) emerges as the state where the analyzed system exhibits the highest LCOW (6.8 and 7.6 $/m^3^) when treating seawater brine and hypersaline brine (70 and 120 g/kg), followed by Rhode Island (RI), Arkansas (AK), Connecticut (CT), Maine (ME), District of Columbia (DC), Massachusetts (MA), and California (CA) (Figure 3e). Although the LCOW in these states surpasses the costs associated with surface discharge and deep-well injection, it remains comparable to evaporation ponds, with California being close to the lowest evaporation cost values. Then, the studied system is a economically viable alternative for the treatment of brine with zero liquid discharge, based on the location conditions. This analysis considers the treatment of 10% of the current produced brine per state but aims to show the potential of the state for producing freshwater from unconventional water sources. The current results indicate the states where is feasible to consider the implementation of brine treatment systems.

For the same studied system, California emerges as the state with the less favorable second-law efficiency when treating seawater brine or hypersaline brine under the base-median case (0.22 and 0.26). Following are Florida (FL), Texas (TX), Oregon (OR), and Washington (WA) (Figure 3b). This is attributed to the less favorable conditions (brine temperature and total brine to treat).

Conversely, New Hampshire (NH) emerges as the state where the analyzed system exhibits the highest second-law efficiency (0.28 and 0.33) when treating seawater brine and hypersaline brine (70 and 120 g/kg), followed by Arkansas (AR), Connecticut (CT), Columbia (DC), Rhode Island (RI), Delaware (DE), Maine (ME), and Montana (MT) (Figure 3d). Although the second-law efficiency in these states surpasses the efficiency of the states with lowest LCOW, the amount of brine treated remains below 0.19 MGD as these states produced low brine having a less developed desalination industry.

In the contiguous United States, second-law efficiency surpasses reported literature values for standalone desalination technologies, all of which remain below 20% efficiency.^31^ This emphasizes the inherent advantages that hybrid designs possess over stand-alone configurations in terms of thermodynamic performance. The remaining states with intermediate results are available in Supporting Information (Figure S7 and Figure S8).

Comparing the economic and thermodynamic potential for treating the brine from the current desalination industry in the US (Figure 3), the correlation between LCOW and second-law efficiency exists in the states with the most favorable LCOW. Texas has the minimum cost for treating brine but also reports the third lowest second-law efficiency. California, on the other hand, reports the lowest second-law efficiency and is part of the highest costs. These results are relevant as California and Texas, along with Florida, are the states that more brine produces currently in the contiguous US (40%, 23%, 13%). Arizona, Oregon, Washington, and Virginia follows with 2% each. As the LCOW depends on location, energy cost, and PV size, optimization is crucial for estimating the desired size of PV-ZLD plants in these relevant states (Figure S9).

### Multiobjective Criteria Per State

**Figure 4:**
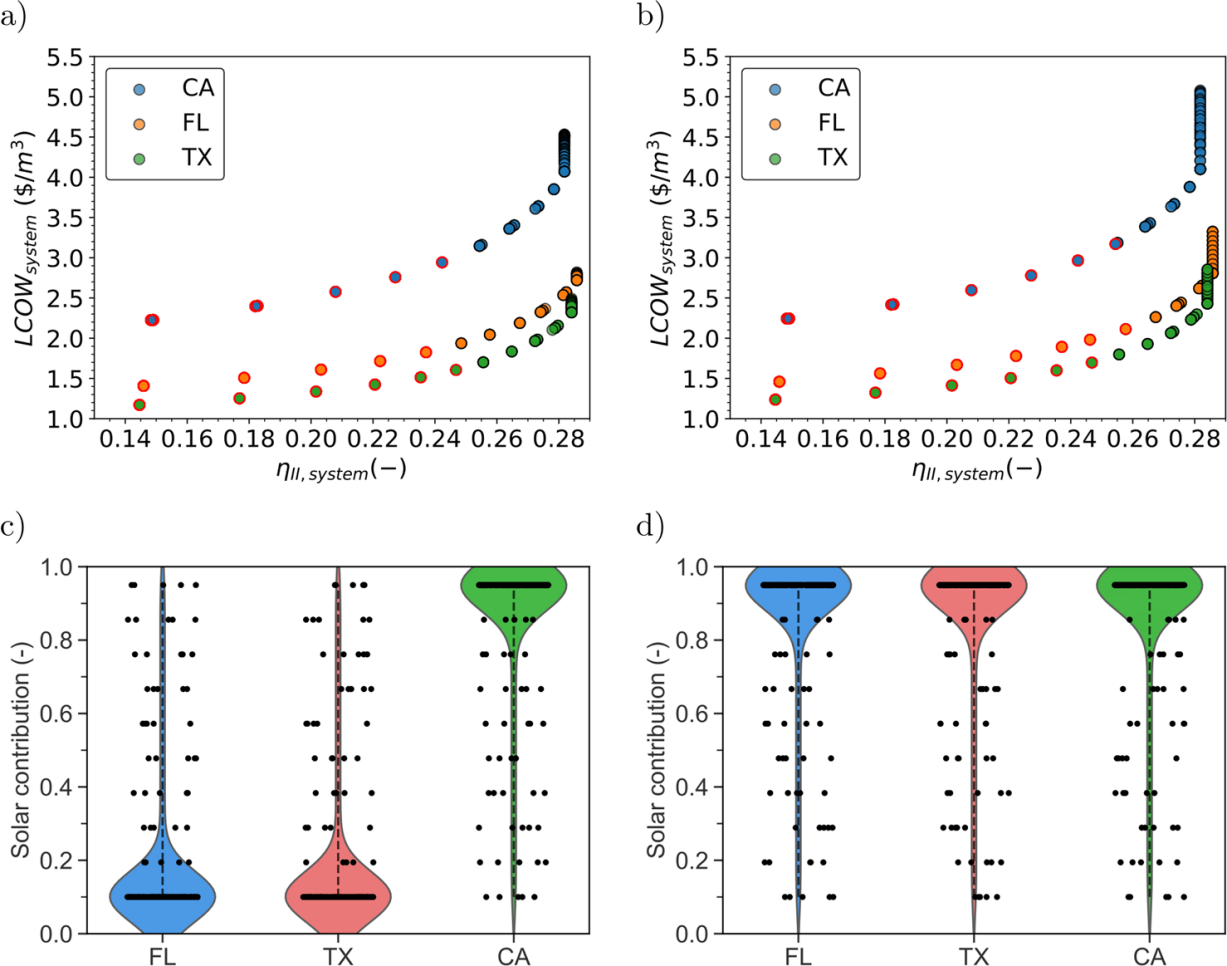
Multiobjective analysis of the studied system applied into California, Texas, and Florida. (a) Pareto front of the defined objective function in the three states of interest. (b) Pareto front of the defined objective function in the three states of interest when considering a carbon Tax. (c, d) Rain cloud plots for the distribution of solar contribution in the Pareto front points without and with carbon tax applied. The red edge dots in panels a and b indicate points where LCOW has a bigger priority in the objective function *f*_obj_.

Under the multiobjective analysis, second-law efficiency varies from 0.14 to 0.28, without being affected by the state analyzed, and is not influenced by carbon tax (Figure 4a and 4b). The most significant difference is associated with the mean ambient temperature per state used in the second-law efficiency estimation (Figure S3) . Conversely, LCOW varies from state to state, with Texas achieving the lowest value for an equal second-law efficiency. This indicates that this state is most favorable for the implementation of the studied system. Without considering a carbon tax, compared with Florida and California, on average, the LCOW in Texas is 15% and 46% lower. The red edge points in the Pareto front represents the point where the preference is given to the LCOW (γ > 0.5). As γ increases, the LCOW decreases in all states to be as low as 1.17, 1.4, and 2.2 $/m^3^ in Texas, Florida and California (Figure 4a). For an equal decision maker’s priority between LCOW and second-law efficiency (γ = 0.5), Texas exhibits the lowest LCOW (1.61 $/m^3^ against 1.8 and 2.94 of FL and CA) and an almost equal second-law efficiency around 0.25. Increasing the priority to second-law efficiency is directly related to prioritize a system that can treat more concentrated brine as the highest second-law efficiency is related with plants treating high concentration streams (Figure S10a) with low capacity (Figure S10c) . Additionally, from the Pareto analysis, when maximizing second-law efficiency, the CO_2_ emissions reach feasible lower values compared with minimizing LCOW (Figure S10b). In terms of design, the second-law efficiency consideration in the multiobjective analysis allows to incorporate into consideration, the capacity of the system (regarding capacity and concentration) and its environmental impact (emissions). It provides value in this study as prioritizing this metric in the optimization model takes into consideration the plant size and the impact of emission (Figure S10). Even when the CO_2_ tax is included in the LCOW equation, the sole minimization of this metric does not prioritize this factor, indicating the need of considering second-law efficiency.

When considering a carbon tax, the LCOW increases in all the studied states, but compared with Florida and California, on average, the LCOW in Texas is 14% and 43% lower. For γ = 1 the system achieves the minimum LCOW with values of 1.24, 1.46, and 2.2 $/m^3^ in Texas, Florida, and California. As expected, the carbon tax increases the LCOW in all states (Figure 4b). For an equal decision maker’s priority between LCOW and second-law efficiency (γ = 0.5), Texas exhibits the lowest LCOW (1.69 $/m^3^ against 2.1 and 3.1 of FL and CA) and almost equal second-law efficiency around 0.25.

Without the carbon tax, the best configurations for the PV-ZLD plant in Texas and Florida, from the Pareto Front, distribute in a region with a solar contribution of 10%, while California has his points distributed around a solar contribution of 95% (Figure 4c and 4d). This is explained by the high price of the grid electricity when compared with the other three locations (0.1482 $/kWh for CA, 0.0765 for FL, and 0.0612 for TX^61−63^). Penalizing the LCOW with a carbon tax increases the potential of the PV system in Florida and Texas, as the solar contribution is 95% alike in California. This is due the fact that increasing the solar contribution will decrease the carbon tax, which influences the decision of the Pareto Front to the ones with the largest solar field.

This analysis not only evaluates the metrics discussed in this work but also offers insights into the conditions exhibited by the studied variables in each state. The examination of the design space provides valuable insights into the system dimensions related to the brine to be treated and the size of the PV field (Figure S11).

The plant capacity in the system’s solution set varies with the state but is not influenced by the carbon tax or solar contribution (Figure S11a and S11b). For Texas and Florida, states with low LCOW and high overall second-law efficiency, two distinct configurations emerge from the set of solutions. Within the concentration range of 25 to 80 g/kg (TX) and 25 to 90 g/kg (FL), the studied system optimally performs (Pareto front solution set) at around 13 MGD. Beyond this range, the optimal plant size is 2 MGD or less. Conversely, for California, the optimal size varies from 0.6 when treating brine with a concentration above 90 g/kg to 2.8 MGD when treating brine with a concentration below 90 g/kg. In all cases, a clear relationship exists between the optimal capacity and the concentration of the PV-ZLD plant. The more concentrated the brine to be treated, the smaller the system in terms of capacity and PV size.

For Texas and Florida, the PV field has a larger size when treating more brine, due to the solar contribution of 95% and due to the fact that as more brine is treated, more power is needed (Figure 4b, Figure S11c, and S11d). Consequently, in these states, a larger system performs better when treating brine with a concentration below 90 g/kg. Conversely, a smaller system is more suitable for treating highly concentrated brine, implying a smaller PV field with a solar contribution ranging from 10% to 95%. Similar trends are observed for California, though with a smaller plant scale. A larger PV size is associated with treating more brine, limited to around 3 MGD. These configurations are effective for treating low salinity brine (less than 70 g/kg). When addressing hypersaline brine, the system size aligns with that of Florida and Texas.

The key finding from this multiobjective analysis suggests that, in states with a low energy grid price, the studied system can compete with traditional brine discharge methods in terms of cost and surpass conventional desalination systems in second-law efficiency. The minimal change in the LCOW from the Pareto front, even when considering a carbon tax, suggests that the grid carbon intensity is not as influential as the grid electricity price. However, it does impact the increase in solar contribution within the Pareto optimal solution set for states with low grid energy prices, such as Texas or Florida.

From the Pareto optimal set per state, decision makers may decide the configuration of the plant based on their preference. In the three states, the system reaches its minimum LCOW, considering the carbon tax, (γ = 1) when the inlet feed has a concentration of 25 g/kg, therefore, for treating brine, the system cannot operate at his minimum cost. However, when treating brine, the system maximizes its second-law efficiency. Regarding capacity, there is an inverse relation between allowable feed flow, LCOW, and second-law efficiency in the Pareto solution set (Figure 4). Then, as more saline is the brine to treat, the optimum capacity is smaller, based on the Pareto solution set, and more plants are needed per state.

As mentioned, the selection of a singular solution depends on the preference of decision-makers between second-law efficiency and LCOW, and on the requirements of brine treatment (flow and concentration). When there is no preference available, knee solutions are attractive to study. In the knee region of a Pareto front, a solution improves a determined goal with a small degradation of other objectives, compared to solutions located far from the knee along the front.^65^ Another alternative, is selecting the closest point to the ideal solution (minimum LCOW and maximum second-law efficiency) based on Euclidean distance.^66^ This section explores the influence of selecting a solution from the closest point, defined as the best point, to an ideal solution in every studied state. The brine feed flow, the concentration of the brine, the yield of the photovoltaic field, and the contribution of the photovoltaic field compose the solution set in this work. We also explore a solution selected from minimum LCOW and maximum second-law efficiency (Table 2).

**Table 2:** Pareto Cases Analysis Results Based on Three Scenarios^a^

State	Scenario	Capacity (MGD) - threshold	Concentration (g/kg)	Max-p-regions clusters (plants)	η_II,system_	LCOWCO2 ($/m^3^)
CA	Best	1.86	67	119	0.24	2.97
CA	Min LCOW	2.87	25	97	0.15	2.24
CA	Max eta	0.63	120	215	0.28	4.10
TX	Best	13.00	78	15	0.25	1.70
TX	Min LCOW	13.00	25	15	0.14	1.24
TX	Max eta	0.63	120	91	0.28	2.43
FL	Best	1.86	67	108	0.25	1.98
FL	Min LCOW	13.00	25	26	0.15	1.46
FL	Max eta	0.63	120	141	0.29	2.81

a Best (closer point to ideal solution), minimum LCOW, and maximum second-law efficiency.

In the closest point region, i.e, the best point scenario, the system configuration varies between California, Florida, and Texas. In California and Florida, the best configuration or scenario for the PV-ZLD, implies a plant treating 1.8 MGD with a concentration of 67 g/kg. Therefore, it is suitable for treating seawater brine. The LCOW under this scenario, with a carbon tax, is 2.97 and 2 $/m^3^, and the second-law efficiency is 0.24. In both cases, the solar contribution is 95% and therefore the most relevant parameter that explains this result is the PV yield achievable in every state (5.9 in CA and 4.8 in FL).

In Texas, the best configuration for the PV-ZLD implies that a plant treated 13 MGD with a concentration of 77.78 g/kg. Therefore, it is suitable for treating more brine with a larger concentration, compared with CA and FL. The LCOW under this scenario is 1.7 $/m^3^, and the second-law efficiency is 0.24. The solar contribution is 95%. As Texas has a PV yield of 5.4 and a grid cost of 6.12 cents/kWh, this state has a greater potential than California and Florida being able to operate with the same efficiency but with a lower cost.

Under the base scenario, none of the locations can treat hypersaline brine. For that, the plant must be smaller (0.6 MGD). However, this is also the configuration that achieves the maximum LCOW in California, Florida and Texas (4.1, 2.8, and 2.4 $/m^3^).

### Identifying the Number of Plants from Multiobjective Analysis

**Figure 5:**
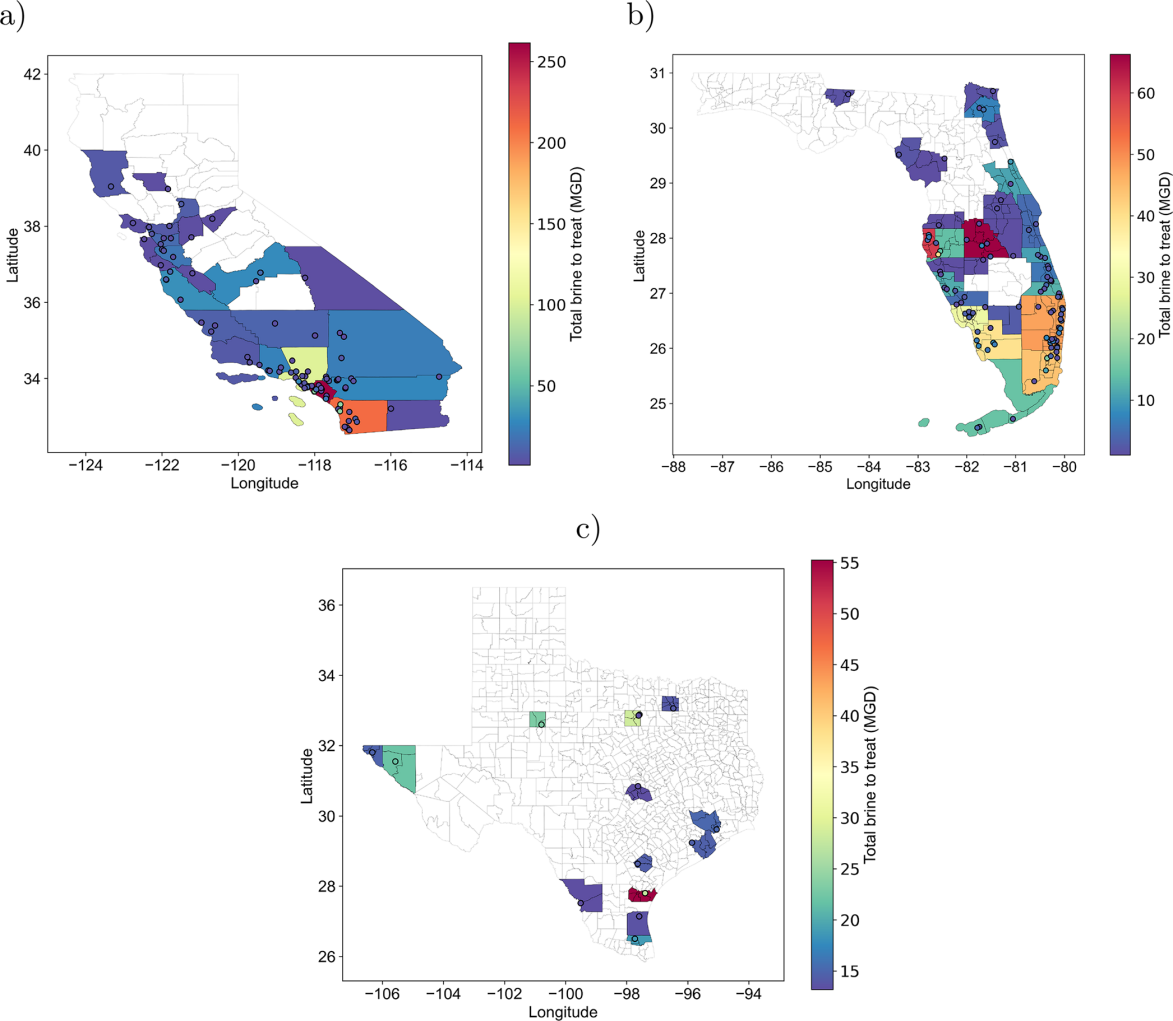
PV-ZLD plant distribution based on centroid per county, using the best case from Multiobjective analysis. The cluster distribution for the required PV-ZLD plants is based on spatially constrained clustering in using the max-p-regions algorithm.^67^ The color bar indicates the amount of brine to treat (MGD) per county inside (a) CA, (b) FL, and (c) TX. White color indicates counties where no plant should be allocated.

Based on the best case scenario per state. It is possible to have a visual representation of the region where the plants might be located, considering the current brine production per state. The estimated brine flow is the desired capacity of the PV-ZLD plant. The number of required plants per region is determined using the max-p-regions algorithm. This clustering algorithm groups the current desalination plants in the regions of interest.^1^ Every cluster is assigned to one PV-ZLD plant. The selected algorithm allows spatially constrained clustering that aggregates areas (polygons) into an unknown number of homogeneous regions ensuring the satisfaction of a minimum threshold value defined from an attribute (in this case, the summation of the brine flow from all the desalination plants belonging to the cluster).^67^ Then, the number of clusters that the algorithm provides is indicative of the number of plants needed in the state and the centroid the tentative region where can be allocated. Every centroid belongs to a county; therefore, it is possible to identify the number of plants and the amount of brine to treat per county. The max-p-regions algorithm uses a threshold value for clustering plants based on the minimum value and aggregates regions (represented by desalination plants in this work) based on their neighbors. However, this algorithm does not provide the optimal location of the ZLD plant but the minimum number of plants needed in the region that fulfills the threshold requirements based on optimal design. Moreover, the cluster are not homogeneous in size or in the amount of brine to treat (Figure 5).

California has 119 centroids (plants) treating from 1 to 77 MGD with a threshold value of 1.86 (Table 2), a median of 2.7 MGD and a mean of 6.8 MGD. The clusters distribute along the territory with a high concentration of plants in the south of California (Figure 5a). This is related with the larger number of plants in the south region (points in Figure 5a) which increases the amount of brine to treat. Comparing the spatially constrained clustering results and the multiobjective analysis results (Table 2), only two plants treat around 1.86 MGD (with a ±10% difference); therefore, the plants are far from the best configuration. On the other hand, 4 plants had a capacity in the range of 2.87 (±10%) which represents the lower LCOW scenario, and none in the range for hypersaline brine treatment.

Florida has 108 centroids (plants) treating from 1 to 39 MGD with a threshold value of 1.86 (Table 2), a median of 2.42 MGD and a mean of 4.15 MGD. The clusters distribute along the territory with a high concentration of plants in middle Florida and south Florida (Figure 5b). This is related with the larger number of plants in these regions. Comparing the spatially constrained clustering and multiobjective analysis results (Table 2), 12 plants treat 1.86 MGD (±10%), i.e, their capacity aligns with the best configuration. On the other hand, none of the plants had a capacity in the range of 13 MGD (±10%), which represents the lower LCOW scenario, and none in the range for hypersaline brine treatment.

Texas has 15 centroids (plants) treating from 13 to 27 MGD with a threshold value of 13 (Table 2), a median of 14.8 MGD and mean of 17.4 MGD. The clusters distribute along the territory and around the borders of the state, with a high concentration of plants southeast Texas (Figure 5c). Comparing the spatially constrained clustering with the multiobjective analysis results (Table 2), 5 plants treat 13 MGD (±10%), i.e., their capacity aligns with the best case configuration and the lower LCOW scenario. On the other hand, none of the plants are in the range for hypersaline brine treatment. The remaining plants in Texas had a capacity ranging from 14 to 27 MGD.

The combination of technoeconomic, multiobjective, and spatially constrained clustering analysis provides insights about the current potential in the US for implementing a PV-ZLD system. Currently the US produces more than 1000 MGD of brine concentrated in three main states (California, Florida, and Texas). When considering a multiobjective approach (including the minimum LCOW and maximum second-law efficiency), Texas shows the biggest potential (followed by Florida), being able to treat seawater brine with a capacity around 13 MGD and with a LCOW comparable with deep-well injection and lower than evaporation ponds. Texas potential arises when identifying the clusters of desalination plants; here, there are clusters far from the coast, making discharge to the ocean not viable. Treating all the brine produced in Texas allows recovery of up to 260 MGD of freshwater. On the other hand, California (the state that produces more brine) is less favorable in terms of cost and second-law efficiency for implementing the studied system. In this region, the best brine management strategy is ocean discharge. The cost improvement in ZLD systems implies a reduction in the concentration entering the first stage (RO system). The mixture of different incoming brine might lead to a decrease in the concentration of the total brine to be treated due to the dilution of high saline brine, consequently reducing the LCOW. From a technological improvement perspective, an increase in the recovery ratio of the RO system operating at high pressure and concentrations might displace the need for MVC systems as a brine concentrator. The use of spatially constrained clustering allows identification of the minimum required number of plants (regions) in the studied states based on the best design.

## Supporting Information Available

Details of the developed computational model and additional figures and tables complementary to manuscript’s results (PDF)
