# Supplementary material for: Electrified Solar Zero Liquid Discharge: Exploring the Potential of PV-ZLD in the US
Source: Environ Sci Technol. 2024 May 3;58(35):15562–74. doi: 10.1021/acs.est.4c00494 (PMC11375782; doi:10.1021/acs.est.4c00494)
Supplement: Supplementary file 1 — es4c00494_si_001.pdf [file es4c00494_si_001.pdf]

Supporting Information:
Electrified Solar Zero Liquid Discharge: Exploring the Potential of PV-ZLD in the US

Rodrigo A. Caceres Gonzalez[1,2] and Marta C. Hatzell[3,4]

This PDF file has 33 pages and contains

1. Fig S1 to S10 (pages S2 to S9)
2. Table S1 to S3 (page S10)
3. Supplemental methods including the computational thermodynamic model and economic model (pages S11 to S26)
4. Solar field model (pages S27 to S29)
5. SI references (pages S29 to S33)

[1]George W. Woodruff School of Mechanical Engineering, Georgia Institute of Technology
[2]School of Industrial Engineering, Faculty of Engineering and Science, Universidad Diego Portales
[3]George W. Woodruff School of Mechanical Engineering, Georgia Institute of Technology
[4]School of Chemical and Biomolecular Engineering, Georgia Institute of Technology

S1 Supplemental figures

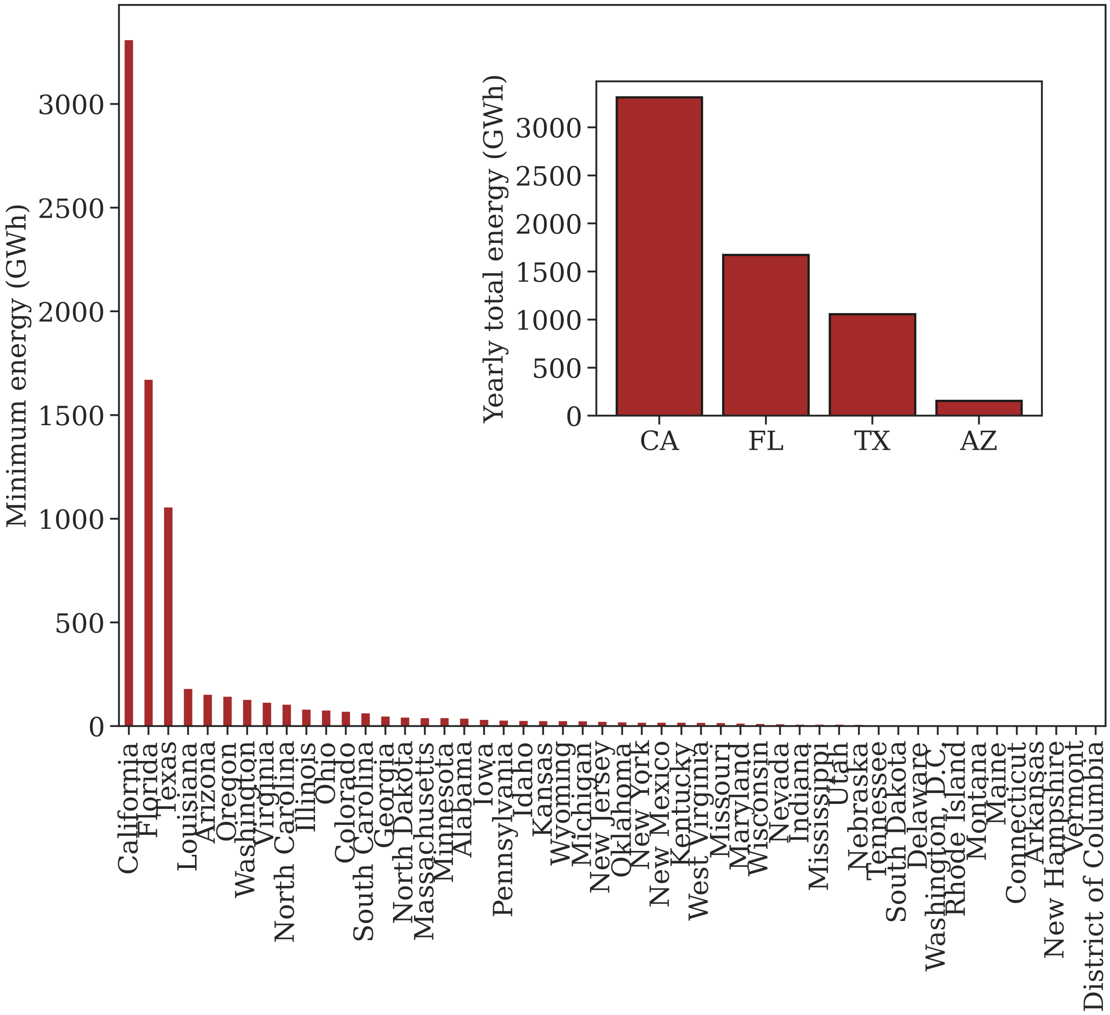

Figure S1: Annual minimum energy of separation required for treating the brine of every desalination plant in the contiguous US. The subplot presents the minimum energy required for the 4 states selected in this work. Louisiana represents a special case since produce less brine than Arizona, but requires more energy.

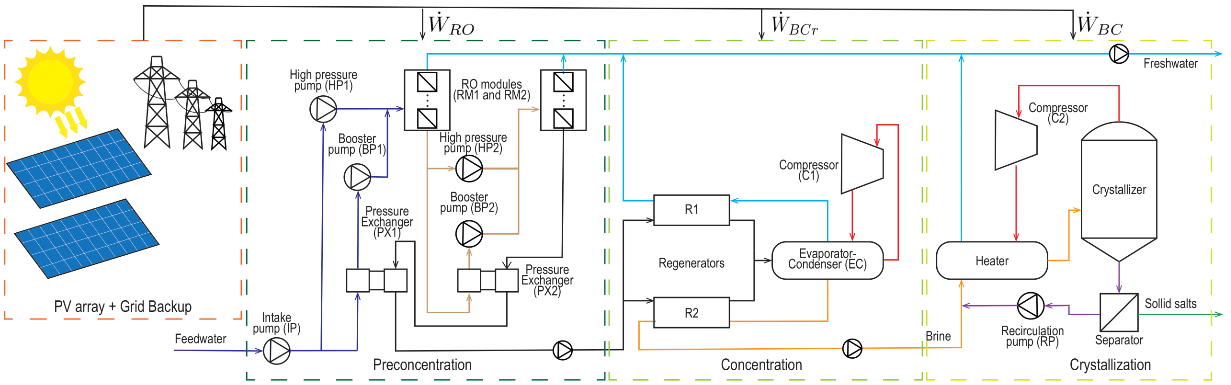

Figure S2: Diagram for the integrated two stages RO (light green dotted box), single-effect MVC (dark green dotted box). and brine crystallizer (light green dotted box) subsystems working as a zero liquid discharge system. The incoming water (feedwater) is preconcentrated in the RO system to be further concentrated until saturation (260000 PPM) in the MVC system. The last step crystallize and separate the salts from the water. A PV system with backup from the electricity grid (orange dotted box) provides the energy required during the process (black line). The total freshwater water produced is the combination of the product streams of every subsystem (light blue line).

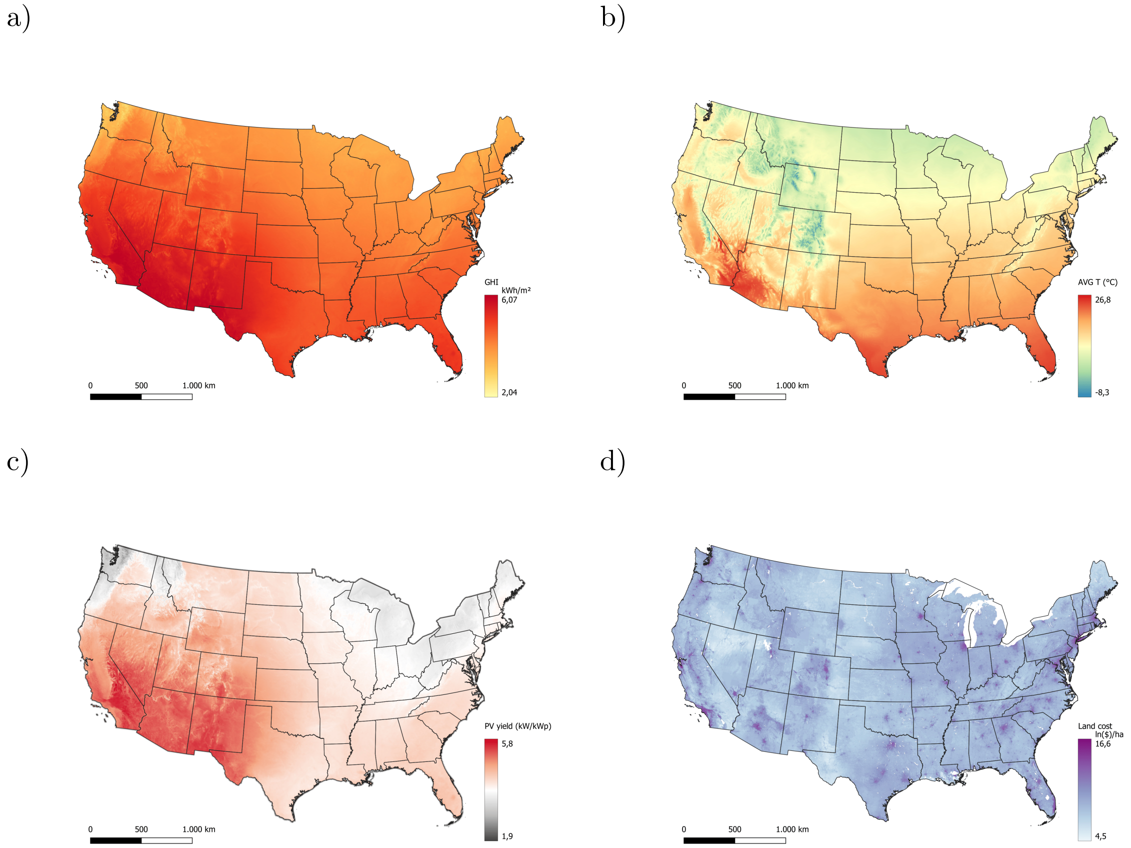

Figure S3: Geographic data for the contiguous US used in the base case in this work in Table **??**. The data represents a) Yearly GHI, b) average Temperature in (°C), c) PV yield and d) land cost.

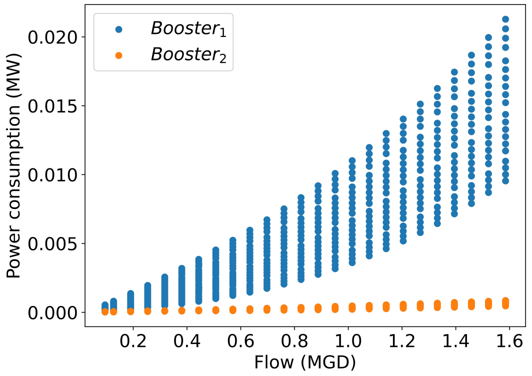

(a) Booster pumps

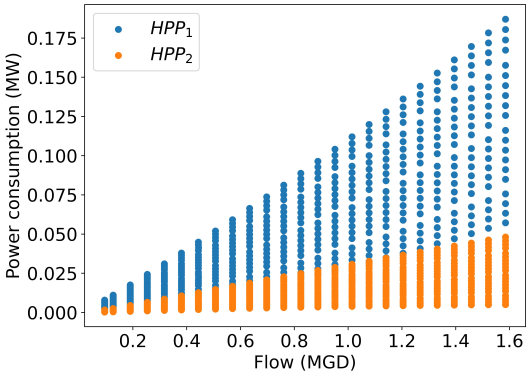

(b) High pressure pumps

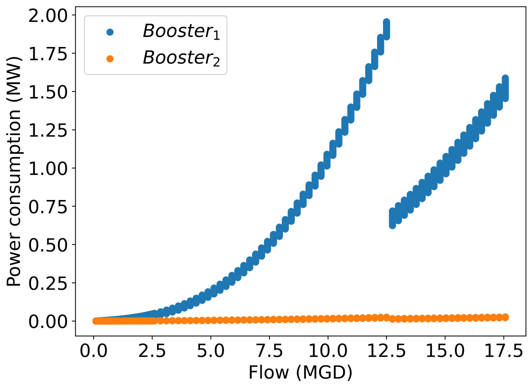

(c) Booster pumps

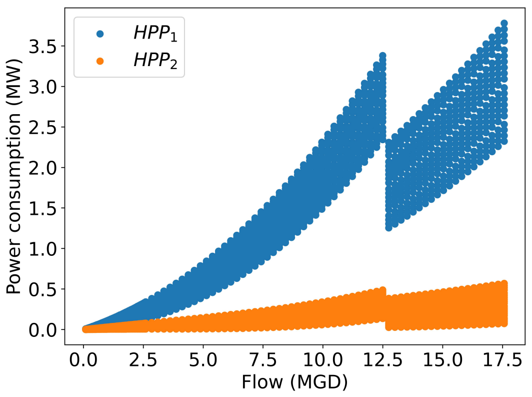

(d) High pressure pumps

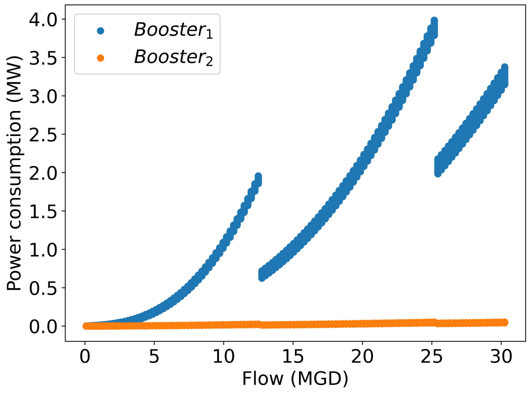

(e) Booster pumps

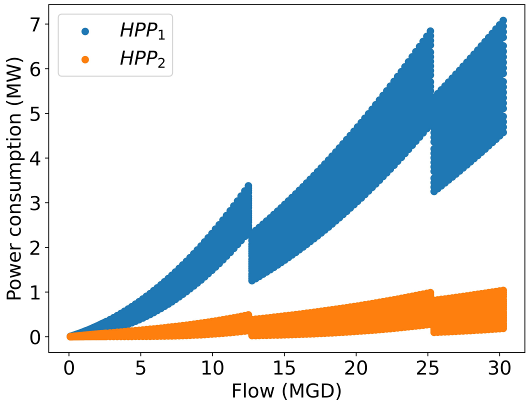

(f) High pressure pumps

Figure S4: Pumping power variation with feed flow for booster and high-pressure pumps inside the RO (preconcentration) subsystem. The threshold value of 12.7 MGD implies the addition of a new unit operating in parallel inside the RO module.

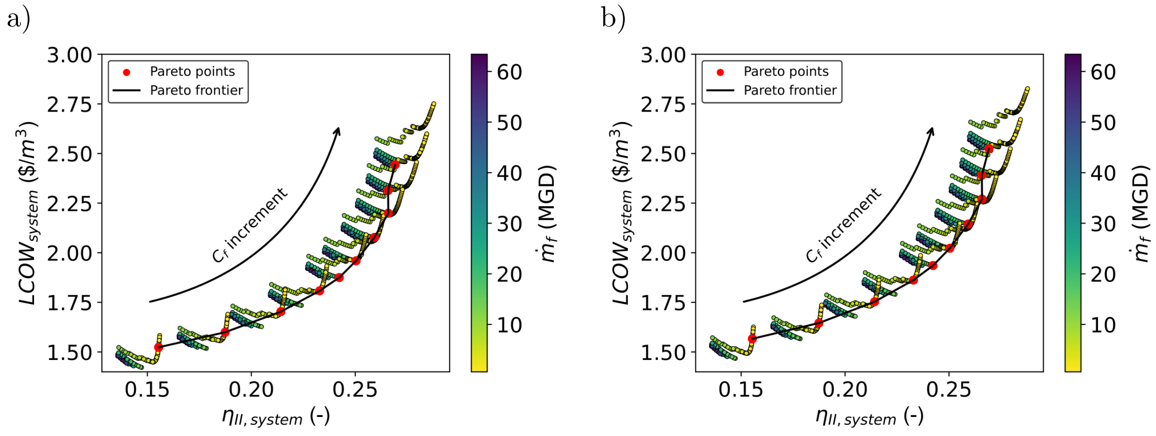

Figure S5: LCOW and second-law efficiency as a function of feed flow and concentration for a PV field contributing to a) 10% of the system power requirements, and b) 90%. The red points and black line represent the Pareto points and front. Colorbar follows the increment in feed flow. Figure 1d compares the Pareto points from the previous cases.

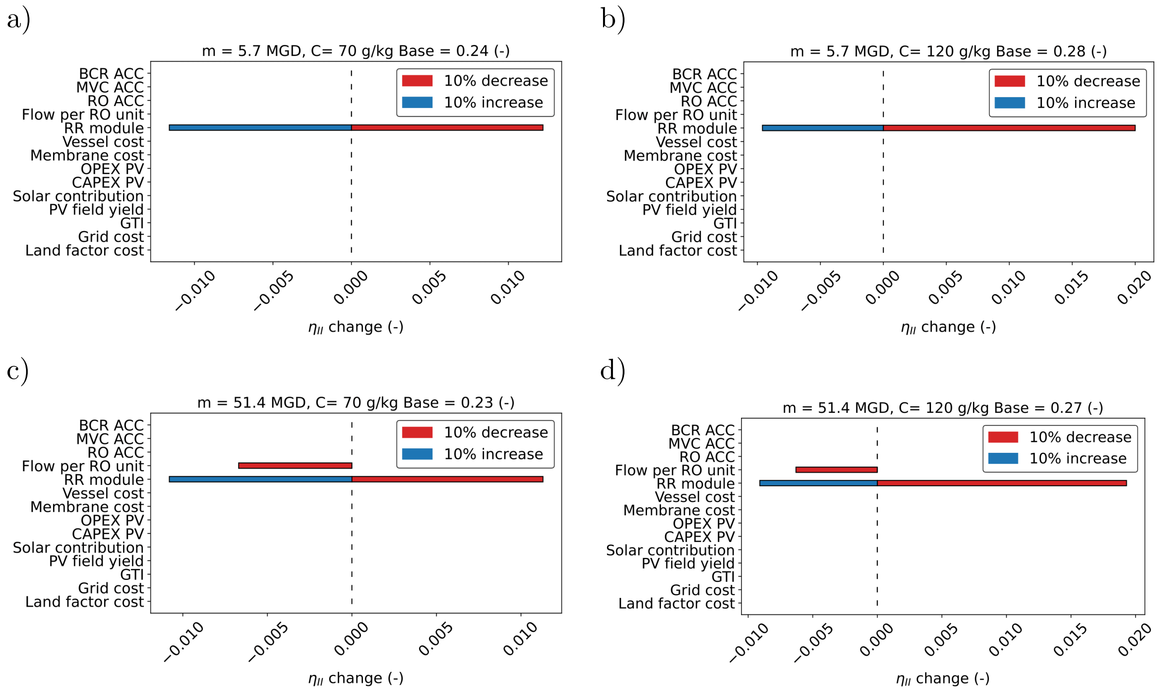

Figure S6: Sensitivity analysis of the ZLD plant second-law efficiency with 50% of solar contribution when varying: a) location related parameters for treating 5.7 MGD at 70 g/kg; b) location related parameters for treating 5.7 MGD 120 g/kg; c) operation and cost parameters for treating 5.7 MGD at 70g/kg; d) operation and cost parameters for treating 5.7 MGD at 120g/kg. Base case values are presented in Table ??. Each parameter varies 10%

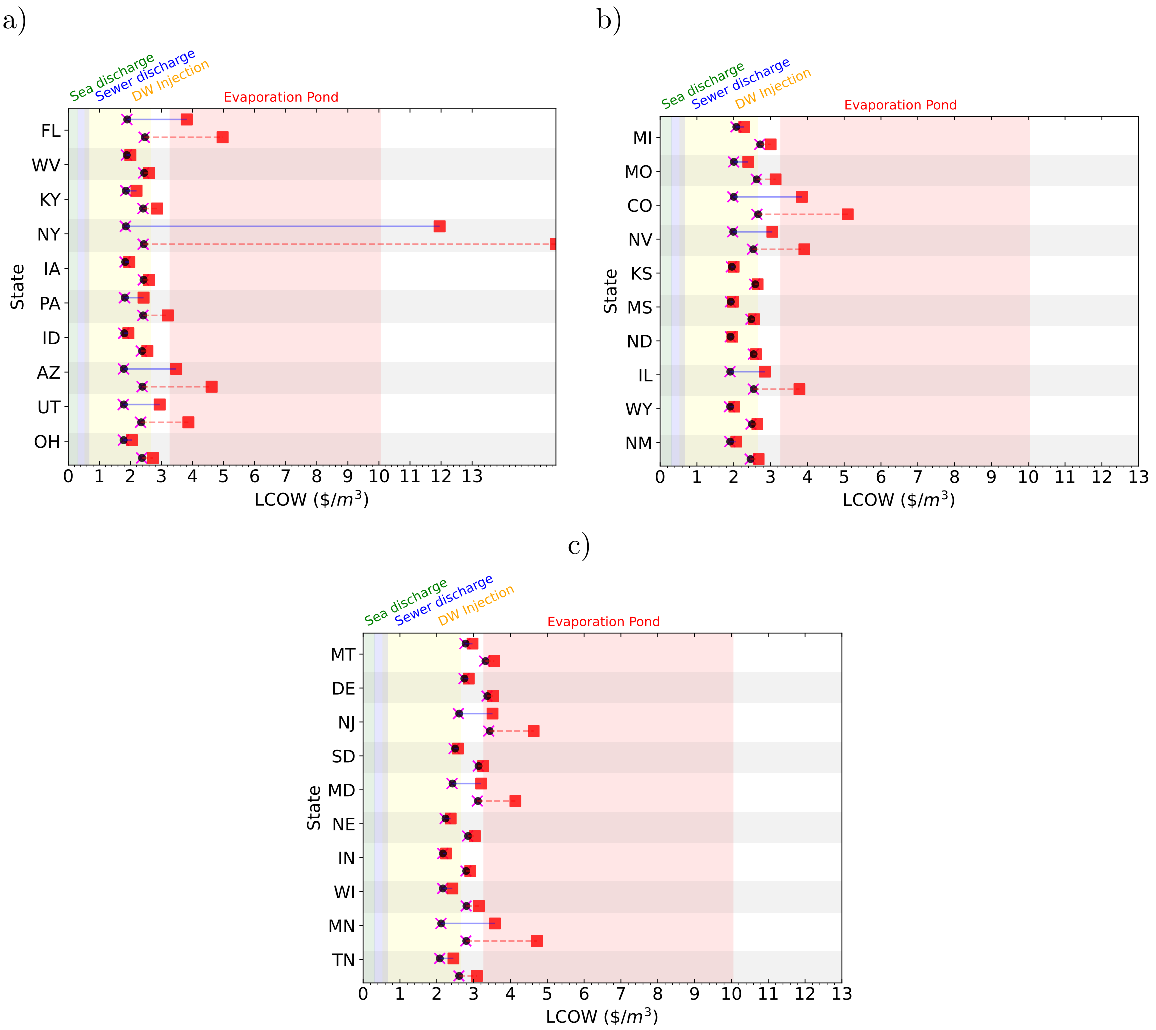

Figure S7: LCOW range for the US when considering a plant treating seawater brine (concentration of 70g/kg) and hypersaline brine (concentration of 120 g/kg) with capacity of 10% the produced brine per state. States are ordered from the lowest LCOW when considering the median geospatial variables considered (PV yield, GTI, ambient temperature, and land cost) to the highest. Figure also shows worst and best scenario of geospatial variables (i.e, lowest land cost and highest solar potential).

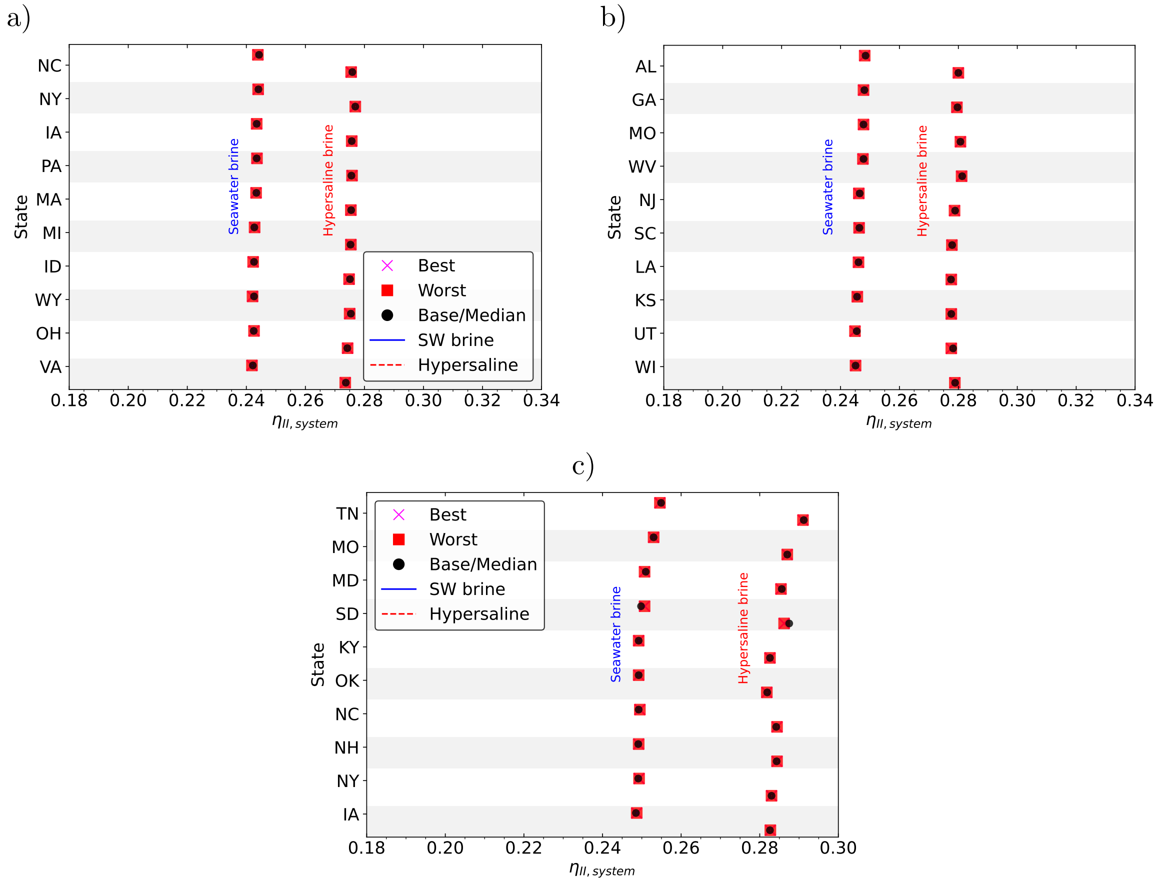

Figure S8: Second-law efficiency range for the US when considering a plant treating seawater brine (concentration of 70g/kg) and hypersaline brine (concentration of 120 g/kg) with capacity of 10% the produced brine per state. States are ordered from the lowest LCOW when considering the median geospatial variables considered (PV yield, GTI, ambient temperature, and land cost) to the highest. Figure also shows worst and best scenario of geospatial variables (i.e, lowest land cost and highest solar potential).

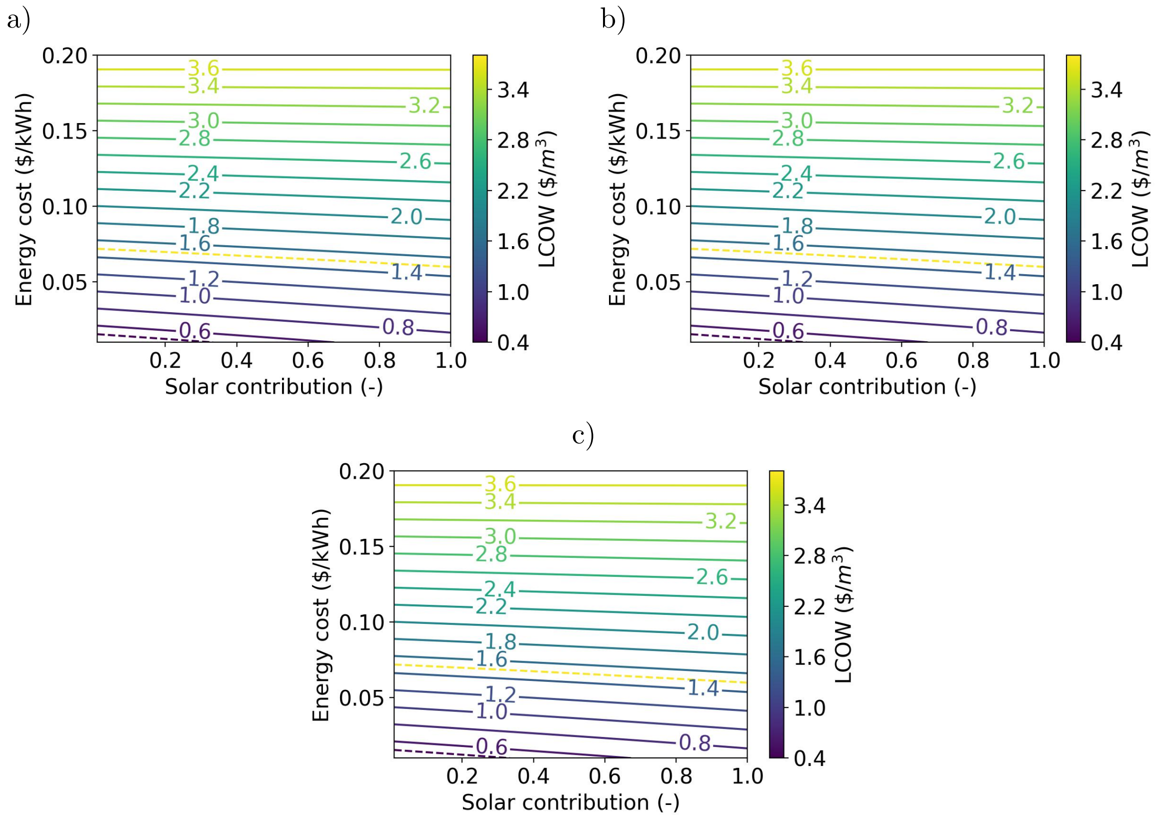

Figure S9: Levelized cost of water sensitivity analysis for changes in PV field size and grid energy cost for base case conditions (Table 1 of main manuscript) in a)California, b) Florida, and c) Texas.

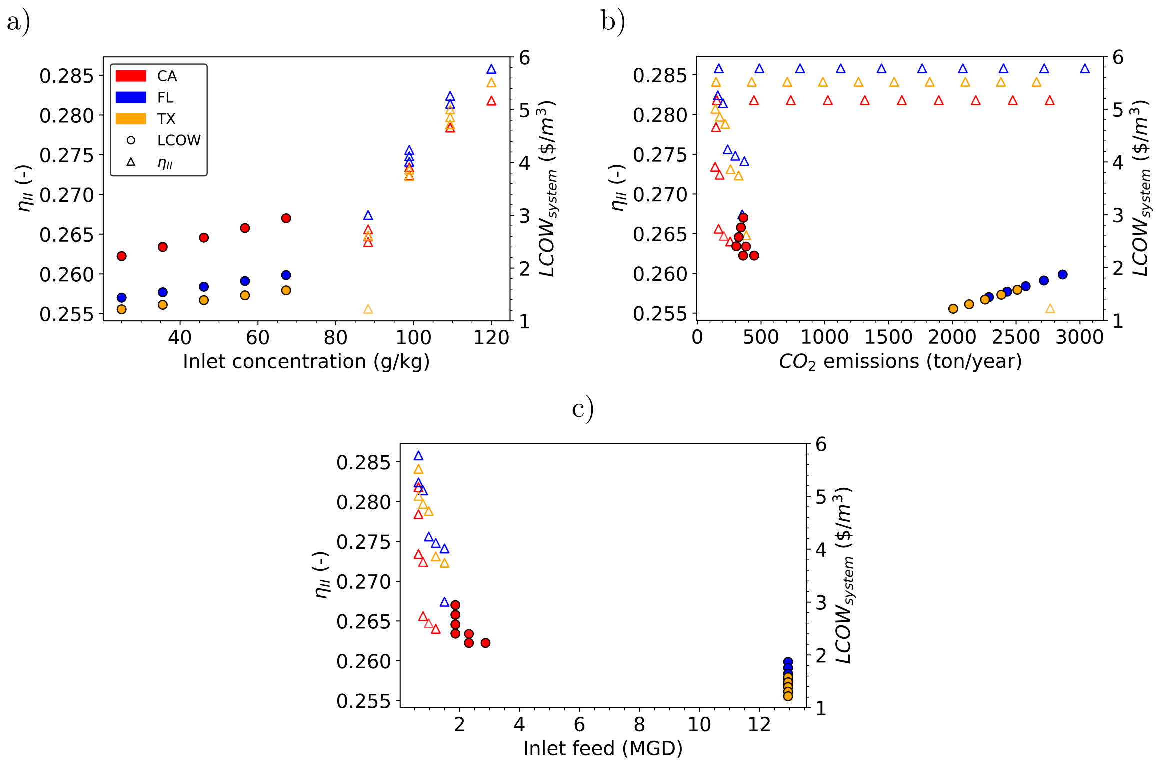

Figure S10: Second-law efficiency and levelized cost influence in a) inlet concentration, b) carbon emissions, and c) inlet flow from the multi-objective analysis. The triangles represent Pareto Point with prioritization of second-law efficiency (> 55% of objective function), and the circles represent Pareto points with prioritization of cost (> 55% of objective function) in California (red), Florida (blue) and Texas (orange).

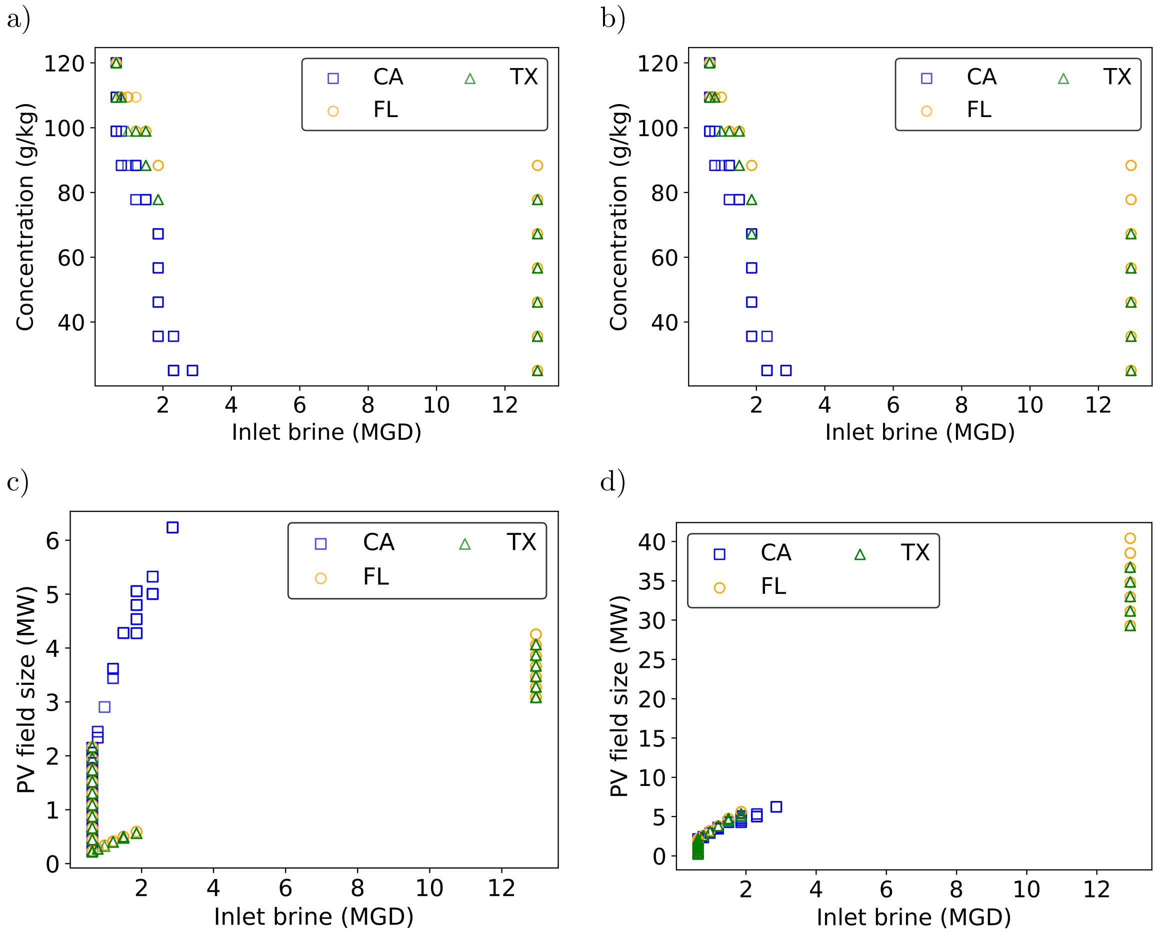

Figure S11: Multi-objective analysis design space for inlet brine versus a) concentration without carbon tax, b) concentration with carbon tax, c) PV field size without carbon tax, and d) PV field size with carbon tax.

S2 Supplemental tables

Table S1: Recovery ratio per technology and feedwater category based on salinity levels[1]

Feedwater	RO	MSF	MED	NF	ED	EDR	Other
Seawater	0.42	0.22	0.25	0.69	0.86	0.4	0.4
Brackish	0.65	0.33	0.34	0.83	0.9	0.9	0.6
Riverwater	0.81	-	0.35	0.86	0.9	0.96	0.6
Purewater[5]	0.86	0.35	-	0.89	0.9	0.96	0.6
Brine	0.19	0.09	0.12	-	0.85	-	0.4
Wastewater	0.65	0.33	0.34	0.83	0.9	0.6	0.6

Table S2: Crystallization subsystem input parameters

Parameter	Unit	Value	Source
Pump efficiency (η_p)	-	0.75	[2, 3]
Compressor efficiency (η_C)	-	0.75	[4, 5]
Crystallization ratio (R_{cr})	-	0.9	[2]
Recirculation rate (R_{rec})	kg/kg	42.38	[2, 3]
Crystallizer circulation pressure drop (ΔP_{cf})	kPa	150	[2]
Temperature pinch point for vapor in heater	K	3	[5]
Salt (NaCl) specific heat	kJ/kgK	0.88	

Table S3: PV panel reference parameters [6]

Parameter	Unit	Value
T_{cref}	°C	25
NOCT	°C	46.4
β	1/°C	-0.0031
η_r	-	0.205521

[5]Purewater refers to water source with low salinity (< 500 PPM) which is desalinated for industrial applications that requires low salinity water like food processing or pharmaceutical applications)

S3 Supplemental methods

S3.1 Computational model of the PV-ZLD system

S3.1.1 RO subsystem

In the RO subsystem, the feed flows through an intake pump before splitting in two parts, one flows directly to the RO module (RM1), while the other to the pressure exchanger (PX1). In every RO module (RM1 and RM2) the permeate flow is[7]

$$m_p = (\Delta P - \Delta\pi) \cdot TCF \cdot FF \cdot n_e \cdot n_v \cdot k_w \cdot A_e \tag{1}$$

where ΔP is the change of pressure inside the RO module, $\Delta\pi$ is the change of osmotic pressure inside the RO module, TCF a temperature correction factor, FF, the membrane fouling factor, n_e the number of membrane elements inside a pressure vessel, n_v the number of pressure vessels in the system, k_w is the membrane water permeability and A_e the area of every membrane element.

ΔP and $\Delta\pi$ compares the average of the feed flow and brine with the permeate as follows

$$\Delta P = \bar{P} - P_p \tag{2}$$

$$\Delta\pi = \bar{\pi} - \pi_p \tag{3}$$

where $\bar{P}$ and $\bar{\pi}$ are the average pressure and osmotic pressure between the inlet stream and brine, while P_p and π_p are the pressure and osmotic pressure of the permeate. The pressure of the brine is equal to the pressure of the flow entering the RO module minus the pressure drop inside the membrane array

$$P_b = P_f - P_{drop} \tag{4}$$

where P_b is the brine pressure, P_f the inlet flow pressure and P_drop the pressure drop inside the membrane element defined as function of the flux crossing the membrane element[8]

$$P_{drop} = 0.01 \cdot n_e \left(\frac{\dot{m}_f + \dot{m}_b}{2n_v} \right)^{1.7} \tag{5}$$

where $\dot{m}_f$ and $\dot{m}_b$ are the inlet and brine flux in GPM. Pressure drop provided by this equation has psi as unit, so proper conversion to kPa is needed.

The temperature correction factor and fouling factor are used for estimate the membrane water permeability considering operation temperatures different than the nominal ones[9]

$$TCF = \exp \frac{e}{R}(\frac{1}{T} - \frac{1}{T_0}) \tag{6}$$

where R is the universal gas constant in $J/mol - K$, T the temperature of the flux in K and T_0 a reference temperature, in this case 298 K. e is the membrane activation energy defined as 25000 J/mol when T $\leq$ 298 and 22000 J/mol in any other case

Table S4 shows the principal parameters for the RO system.

The salt transport through the membrane array is function of the membrane salt permeability and the concentration polarization

$$m_s = (C_w - C_p) \cdot k_s \cdot A_e \cdot n_e \cdot n_v \tag{7}$$

where m_s is the transport of salts, C_w the wall mass salts concentration in kg of salts per kg of water, C_p the concentration of the permeate, k_s the membrane salt permeability in kg/m^2-S, which is function of the fouling factor and temperature correction factor as follows [7]

$$k_s = FF \cdot TCF \cdot 4.72 * 10^{-7}(0.06201 - (5.31 * 10^{-5} \cdot T)) \tag{8}$$

The wall mass salts concentration depends on the recovery ratio and the feed, brine, and permeate concentrations as follows

$$C_w - C_p = \left(\frac{C_f + C_b}{2} - C_p \right) \cdot \exp\left(K \cdot RR\right) \tag{9}$$

where RR is the recovery ratio and K=0.7 [9]

In the pressure exchangers (PX1 and PX2) the feed exchange pressure with the brine for de-

creasing the pumping energy consumption of the high pressure pumps. The recovered pressure depends on the pressure exchanger efficiency, flow densities and pressures as follow

$$P_{rec} = P_{L,in} + \eta_{PX} \cdot \left(\frac{\rho_L}{\rho_H}\right)(P_{H,in} - P_{H,out}) \tag{10}$$

where P_{rec} is the recovered pressure or the pressure of the low-pressure fluid leaving the device the device, $P_{L,in}$ if the pressure of the low-pressure fluid entering the device. eta_{PX} is the efficiency of the pressure exchanger assumed as 0.96[5]. ρ_L and ρ_H are the densities of the low and high-pressure fluids. $(P_{H,in} - P_{H,out})$ is the pressure difference of the high-pressure fluid along the pressure exchanger.

The pressure exchanger process involves some mix between the high and low-pressure flux. Since they differ in concentration, a volumetric mixing, during the pressure exchange process, increases the salinity of the flux entering the RO module

$$C_{f,out} = C_{f,in}(1 + RR \cdot VM \cdot 1.04) \tag{11}$$

where $C_{f,o}$ is the increased concentration of the fluid leaving the pressure exchanger due the volumetric mixing VM defined as [10]

$$VM = \frac{C_{b,out} - C_{f,in}}{C_{b,in} - C_{f,in}} \tag{12}$$

The pumping power requirements in kW depends on the flux through the device and the pressure change

$$\dot{W}_{pump} = \frac{m_f \cdot \Delta P}{\eta_p} \tag{13}$$

where m_f is the flow along the device in m^3/s, ΔP the change of pressure of the flow and η_p the pump efficiency assumed as 0.85[5].

$$\dot{W}_{RO} = \dot{W}_{raw,pump,1} + \dot{W}_{HP,pump,1} + \dot{W}_{booster,pump,1} + \dot{W}_{HP,pump,2} + \dot{W}_{booster,pump,2} \tag{14}$$

Costs

From literature correlations it is possible to estimate the capital cost of the devices in the RO

subsystem [7, 9, 11]. The total investment of this system considers pretreatment system, pumps, pressure exchangers and RO modules as follows

$$\begin{aligned} CC_{RO} = CC_{SWIP} + CC_{HPP,1} + CC_{HPP,2} \\ + CC_{BP,1} + CC_{BP,2} + CC_{PX,1} + CC_{PX,2} \\ + N_{units} \cdot (CC_{RO,module,1} + CC_{RO,module,2}) \end{aligned} \tag{15}$$

where CC_{SWIP} is the capital cost of pretreatment and intake pumping; $CC_{HPP,1}$ and $CC_{HPP,2}$ the capital cost for both high-pressure pumps; $CC_{BP,1}$ and $CC_{BP,2}$ the capital cost for the booster pumps located outside the pressure exchangers; $CC_{PX,1}$ and $CC_{PX,2}$ the capital cost of the pressure exchangers, $N_{unit}$the number of RO units with 43 vessels and 7 membrane element required for achieving a flow of 2000 m^3/h in every array, and $CC_{RO,module,1}$ and $CC_{RO,module,2}$ the capital cost of every RO module composed by 43 pressure vessels and 7 membrane elements each.

The cost of pretreatment and intake pumping depends on the flow inside the device [11]

$$CC_{SWIP} = 996 \cdot (m_f)^{0.8} \tag{16}$$

where m_f is the flow in m^3/d. The pump capital cost depends on the flux in m^3/h through the device and the outlet pressure desired as follows

$$CC_{pump} = \begin{cases} 52 \cdot m_f \cdot P_f & m_f \leq 200m^3/h \\ 81 \cdot (m_f \cdot P_f)^{0.96} & m_f \leq 450m^3/h \\ 393000 + 10710P_f & a.o.c \end{cases} \tag{17}$$

where P_f is the outlet pressure of the pump in bar. The pressure exchanger capital cost is a direct function of the high-pressure flow entering the device[12]

$$CC_{PX} = 3134.7 \cdot (Q_{HP,in})^{0.58} \tag{18}$$

where $Q_{HP,in}$ is the flux of the high-pressure fluid entering the exchanger in m^3/h. The cost of

every RO module depends on the cost of the membrane and vessel

$$CC_{RO,module} = n_e \cdot n_v \cdot c_{me} + n_v \cdot c_v \tag{19}$$

where c_{me} and c_v are the membrane element and pressure vessel costs (\$1200 and \$1000)[13].

The annual operational cost of the RO subsystem considers power cost, labor, chemicals use, insurance and maintenance as follows [9]

$$OC_{power,RO} = f_c \cdot energy_{cost} \cdot \dot{W}_{RO} \cdot hours \cdot 365 \cdot \tag{20}$$

$$OC_{labor,RO} = f_c \cdot 0.01 \cdot m_p \cdot hours \cdot 365 \tag{21}$$

$$OC_{chem,RO} = f_c \cdot 0.04 \cdot m_p \cdot hours \cdot 365 \tag{22}$$

$$OC_{insur,RO} = 0.005 \cdot CC_{RO} \cdot CRF \tag{23}$$

$$OC_{main,RO} = 0.003 \cdot CC_{RO} \cdot CRF \tag{24}$$

where f_c is the capacity factor of the system assumed as 0.9[14], $energy_{cost}$ is the electricity price,$\dot{W}_{RO}$ is the summation of the pumping power required by the RO subsystem, m_p the produced freshwater from the RO subsystem, and CRF the capital recovery factor used for annualizing the capital costs of the subsystem.

$$CRF = \frac{i \cdot (1+i)^{LF}}{(1+i)^{LF} - 1} \tag{25}$$

where i is the interest rate assumed as 0.05 and LF the lifetime of the plant, assumed as 20 years [7].

Entropy generation

The entropy generation of the RO subsystem is the summation of every device entropy generation.

$$\begin{aligned}\dot{S}_{gen,RO} &= \dot{S}_{gen,RP} + \dot{S}_{gen,HHP,1} + \dot{S}_{gen,HPP,2} \\ &\quad + \dot{S}_{gen,BP,1} + \dot{S}_{gen,BP,2} + \dot{S}_{gen,PX,1} + \dot{S}_{gen,PX,2} \\ &\quad \dot{S}_{gen,RO,module,1} + \dot{S}_{gen,RO,module,2}\end{aligned} \tag{26}$$

where $\dot{S}_{gen,RP}$, $\dot{S}_{gen,HPP,1}$, $\dot{S}_{gen,HPP,2}$, $\dot{S}_{gen,BP,1}$, and $\dot{S}_{gen,BP,2}$ are the entropy generation of the raw pump, first high-pressure pump, second high-pressure pump, first booster pump and second booster pump. $\dot{S}_{gen,PX,1}$ and $\dot{S}_{gen,PX,2}$ are the entropy generation of the pressure exchangers. $\dot{S}_{gen,RO,module,1}$ and $\dot{S}_{gen,RO,module,2}$ are the entropy generation of every RO module.

The entropy generation of every pump is function of the efficiency of the device and the change of pressure as follows [15]

$$\dot{S}_{gen,pump} = \frac{\Delta P}{\rho \cdot T} \cdot \left(\frac{1}{\eta} - 1\right) \tag{27}$$

where $DeltaP$ is the change in pressure of the fluid inside the pump, ρ the fluid density, T the flow temperature entering the pump and η the pump efficiency. Pressure exchanger entropy generation is estimated as an irreversible expansion of an incompressible fluid for the brine and as a pump for the feed [15]

$$\dot{S}_{gen,PX} = \frac{\Delta P_{brine}}{\rho_b \cdot T_b} \cdot (1 - \eta_e) + \frac{\Delta P_{feed}}{\rho_f \cdot T_f} \cdot \left(\frac{1}{\eta_c} - 1\right) \tag{28}$$

where the subscript f and b represents the feed and brine in the pressure exchanger. η_c and η_e are the efficiency of the compression and expansion process assumed as 0.98 [15]. Ro module entropy generation considers the change in composition of the saline water and the depressurization of the permeate and brine streams

$$\begin{aligned}\dot{S}_{gen,module} &= m_p \cdot s_p + m_b \cdot s_b - m_f \cdot s_f \\ &\quad + \left(\frac{(P_f - P_b)}{\rho_b \cdot T_0}\right) \cdot m_b \\ &\quad + \left(\frac{(P_f - P_p)}{\rho_p \cdot T_0}\right) \cdot m_p\end{aligned} \tag{29}$$

Table S4: RO module main parameters [7, 9, 13, 15]

Parameter	Units	Value
FF	-	0.85
n_e	-	7
n_v	-	43
A_e	m^2	35.4
k_w	kg/m^2-s-Pa	2.7x10^{-9}
c_{me}	\$	1200
c_v	\$	1000
η_{PX}	-	0.98
η_c	-	0.98
η_e	-	0.98

S3.1.2 MVC subsystem

In the MVC subsystem, the brine from the RO subsystem (feed for the MVC subsystem), flows through a preheater where exchanges heat with the distilled water and the brine leaving the Evaporator/Condenser device. In the preheater the feed heats until the evaporator temperature (60°C)

$$m_f \cdot c_{p,evap} \cdot (T_{evap} - T_f) = m_d \cdot c_{p,p} \cdot (T_d - T_{out}) + m_b \cdot c_{p,b} \cdot (T_b - T_{out}) \tag{30}$$

where m_f if the brine from the RO subsytem at a temperature T_f and $c_{p,evap}$ is the specific heat of the saline water at the evaporator temperature T_{evap}. m_d, $c_{p,p}$, m_b and $c_{p,b}$ are the mass flow and specific heat of the distillate and brine leaving the evaporator/condenser at temperature T_d and T_b. After the heating process, both distillate and brine leaves the preheater at a lower temperature T_out. To achieve this temperature, the feed is divided in two parts for exchanging heat with the brine and the distilled from the evaproator/condenser. The log-mean temperature differences allows for estimating the heat transfer area of every preheater (or regenerator)

$$\dot{Q}_{PH} = U_{PH} \cdot LMTD_{PH} \cdot A_{PH} \tag{31}$$

where $LMTD_{PH}$ is the log-mean temperature difference between the fluids inside the heat exchanger, A_{PH} the heat transfer area and U_{PH} the overall heat transfer coefficient assumed as 1.185 kW/m^2-K [4].

The compressor suction temperature is lower than the evaporation temperature by the boiling point elevation

$$T_{C,in} = T_{evap-BPE_f} \tag{32}$$

where BPE_f is the boiling point elevation of the feed. The temperature of condensation of the vapor leaving the compressor and flowing into the condenser considers a terminal temperature difference [5]

$$T_{cond} = T_{evap} + TTD \tag{33}$$

where the terminal temperature difference TTD is 3 K. The brine temperature leaving the system is higher than the compressor suction temperature by the boiling point elevation

$$T_b = T_{evap} + BPE_b - BPE_f \tag{34}$$

where BPE_b is the boiling point of the brine at concentration C_b.

The power consumption of the compressor if function of the pressure change in the device and the suction temperature as follows [5]

$$\dot{W}_C = \frac{m_v \cdot c_{p,v} \cdot T_{C,in}}{\eta_c} \cdot \left(PR^{\frac{\gamma_v - 1}{\gamma_v}} - 1 \right) \tag{35}$$

where m_v and $c_{p,v}$ are the flow and specific heat of the vapor in the compressor at the suction temperature $T_{C,in}$. PR is the pressure ratio in the compressor and eta_c the compressor efficiency assumed as 0.7[5].

The log-mean temperature differences allows for estimating the heat transfer area of the evaporator/condenser

$$\dot{Q}_{EC} = U_{EC} \cdot LMTD_{EC} \cdot A_{EC} \tag{36}$$

where $LMTD_{EC}$ is the log-mean tempearture difference between the fluids inside the heat exchanger, A_{EC} the heat transfer area and U_{EC} the overall heat transfer coefficient assumed as 2.56 kW/m^2-K [4].

Costs

From literature correlations it is possible to estimate the capital cost of the devices in the MVC subsystem [4]. The total investment of this subsystem considers preheaters, evaporator/condenser and compressor as follows

$$CC_{MVC} = CC_{PH} + CC_C + CC_{EC} \tag{37}$$

where CC_{PH}, CC_C and CC_{EC} are the capital costs for the preheaters/regenerators, compressor, and evaporator/condenser.

The capital cost of the preheater is function of the heat transfer area[4]

$$CC_{PH} = 1000 \cdot (12.86 + A_{PH}^{0.8}) \tag{38}$$

where A_{PH} is the heat transfer area in m^2. The evaporator/condenser is a tube and shell heat exchanger which capital cost depends on the heat transfer coefficient, area and pressure drop

$$CC_{EC} = 430 \cdot (0.582 \cdot U_{EC} A_{EC} \Delta P_t^{-0.1} \Delta P_s^{-0.1}) \tag{39}$$

where $DeltaP_t$ and ΔP_s are the pressure drop in the tube and shell, assumed as 100 kPa [4]. The capital cost of the compressor depends on the pressure ratio and device efficiency

$$CC_C = 7364 \cdot m_v \cdot PR \cdot \left(\frac{\eta_c}{1-\eta_c}\right)^{0.7} \tag{40}$$

The annual operational cost of the MVC subsystem considers power cost, labor, chemicals use, insurance and maintenance as follows [9]

$$OC_{power,MVC} = f_c \cdot energy_{cost} \cdot \dot{W}_C \cdot hours \cdot 365 \cdot \tag{41}$$

$$OC_{labor,MVC} = f_c \cdot 0.01 \cdot m_d \cdot hours \cdot 365 \tag{42}$$

$$OC_{chem,MVC} = f_c \cdot 0.04 \cdot m_d \cdot hours \cdot 365 \tag{43}$$

$$OC_{insur,MVC} = 0.005 \cdot CC_{MVC} \cdot CRF \tag{44}$$

$$OC_{main,MVC} = 0.003 \cdot CC_{MVC} \cdot CRF \tag{45}$$

where f_c is the capacity factor of the system assumed as 0.9, $energy_{cost}$ is the electricity price, m_d the produced freshwater from the MVC subsystem, and CRF the capital recovery factor used for annualizing the capital costs of the subsystem.

Entropy generation

The entropy generation of the MVC subsystem is the summation of every device entropy generation

$$\dot{S}_{gen,MVC} = \dot{S}_{gen,PH} + \dot{S}_{gen,EC} + \dot{S}_{gen,C} \tag{46}$$

where $\dot{S}_{gen,PH}$, $\dot{S}_{gen,EC}$, and $\dot{S}_{gen,C}$ are the entropy generation of the preheater, evaporator/condenser and compressor.

For the preheater/regenerator, an entropy balance, relating the entropy change of every incompressible fluid inside the device, provides the entropy generation as follows

$$\dot{S}_{gen,PH} = m_b \cdot c_{p,b} \cdot \ln\left(\frac{T_{out}}{T_b}\right) + m_d \cdot c_{p,d} \cdot \ln\left(\frac{T_{out}}{T_d}\right) + m_f \cdot c_{p,f} \cdot \ln\left(\frac{T_{evap}}{T_f}\right) \tag{47}$$

For the evaporator/condenser and entropy balance considering the change of composition in the feed, brine and produced water provides the entropy generation

$$\dot{S}_{gen,EC} = \underbrace{m_d \cdot s_{evap,out} + m_b \cdot s_b - m_f \cdot s_{evap,in}}_{\Delta S_{composition}} + \underbrace{m_d \cdot (s_d - s_{cond,out})}_{\Delta S_{vapor}} \tag{48}$$

where the first term in the expression represents the entropy change due the change on composition of the salt water in the system and the second term the change on entropy of the vapor when condenses into the produced water. The specific entropy and enthalpy of the saline water is function of temperature and concentration and has been correlated in literature[16, 17, 18, 19, 20].

The entropy generation of the compressor is a function of its efficiency and the pressure ratio as follows [15]

$$\dot{S}_{gen,C} = c_{p,v} \cdot \ln\left[1 - \frac{1}{\eta_c} \cdot \left(1 - PR^{\frac{R}{M_v \cdot c_{p,v}}}\right)\right] - \frac{R}{M_v} \cdot \ln(PR) \tag{49}$$

Table S5: MVC subsystem main parameters [4, 5, 9, 13, 15, 21, 22, 23, 24, 25]

Parameter	Units	Value
T_{evap}	°C	60
η_c	-	0.7
U_{PH}	kW/m^2-K	1.185
U_{EC}	kW/m^2-K	2.56
ΔP_t	kPa	0.35
ΔP_s	kPa	0.06

Table S6: Validation table for RO subsystem

Variable	Units	Nafey et al.[7]	ROSA[7]	Current work	Diff-Nafey (%)	Diff-ROSA (%)
m_f	m^3/h	485.9	458.9	486	NA	NA
SEC	kWh/m^3	7.68	7.76	7.97	4%	3%
Pump	kW	1131	1131.42	1162	3%	3%
C_b	g/kg	64	62	64.15	0%	3%
m_b	m^3/h	340.1	340.15	340.2	0%	0%

where R is the universal gas constant, M_v the molar mass of water, PR the pressure ratio, $c_{p,v}$ the specific heat of the vapor and η_c the efficiency of the compressor.

S3.1.3 Validation

The lack of experimental data or real operation values for a large-scale application of the studied hybrid system hinders the validation process. However, it is possible to compare the performance of every subsystem in small-scale operation with values provided by literature (table S6 and S7).

S3.1.4 Brine crystallization subsystem

In the crystallization step, the saturated brine from the MVC subsystem mixes with a recirculation slurry flowing into a heater reaching the saturation temperature exchanging heat with the vapor from the crystallization. The vapor condenses producing freshwater while the heated brine enters

Table S7: Validation table for MVC subsystem

Variable	Units	Chung et al.[26]	Current work	Difference (%)
m_f	kg/s	1	1	NA
C_f	g/kg	35	35	NA
C_b	g/kg	250	250	NA
η_c	NA	0.7	0.7	NA
T_{evap}	K	346	346	NA
TTD	K	3	3	NA
SEC	kj/kg	78.8	86.7	9%
η_{II}	-	8.5	7.7	-10%

the crystallizer. The brine flashes in the crystallizer producing vapor and a high-concentration slurry with salt crystals. The slurry flows into a separator recovering the solid salt while the remaining slurry enters into a recirculation pump. The recirculation ensures a large concentration and operation in the crystallizer. The salt crystal separation process is assumed reversible (no entropy generation)[27].

The brine recirculation flow ratio is constant at 42.38 kg/kg [2, 3] for ensuring high salinity in the crystallizer. For a steady state operation, the saturated brine flow entering the system is equal to the fresh and salt produced. The energy, mass and concentration balances solved simultaneously provides the temperature of vapor and slurry. The input parameters of the model define the performance of the devices (S2)

Energy and concentration balances

In the heater, the vapor coming from the crystallizer condenses transfering heat to the mixed stream between the saturated brine from the concentrator and the recirculating brine from the separator.

$$\dot{m}_{cf} \cdot (h_{cf,out} - h_{cf,in}) = \dot{m}_v \cdot (h_{v,in} - h_{v,out}) \tag{50}$$

where h_{cf} and h_v are the enthalpy of the brine and vapor entering the heater. There is not change in concentration inside this device.

In the crystallizer, the concentration of the brine increases as evaporation occurs, the flow that does not evaporates became a slurry

$$\dot{m}_{cf} \cdot C_{cf} = Slurry \cdot C_{sl} \tag{51}$$

The slurry is equal to the difference between the brinw flow entering the crystallizer and the vapor extracted in the crystallizer.

$$Slurry = \dot{m}_{cf} - \dot{m}_v \tag{52}$$

For conservation of mass in the system, the vapor produced in the crystallizer is equal to the pure water portion of the feed entering the subsystem (brine from the MVC subsytem).

$$\dot{m}_v = \dot{m}_b \cdot (1 - C_b) \tag{53}$$

The amount of salt produced depends on the crystallization ratio and the saturation concentration of the brine inside the crystallizer

$$\dot{m}_s = Slurry \cdot R_{cr} \cdot (C_{sl} - C_{sat})/1000 \tag{54}$$

here, C_{sat} (g/kg) is the saturation of the brine inside the crystallizer which is function of temperature [2]

$$C_{sat} = (0.2628 + 62.75 \cdot 10^{-6} \cdot T_{cb} + 1.08 \cdot 10^{-6} \cdot T_c b^2) \cdot 1000 \tag{55}$$

where T_{cb} is the temperature in the crystallizer and 1000 is a correction factor for converting kg/kg to g/kg

The compressor model is identical to the one in the concentration subsystem [5]. The compressor suction temperature is lower than temperature of the brine in the crystallizer by the boiling point elevation

$$T_{C,s} = T_{cb} - BPE_{cf} \tag{56}$$

where BPE_{cf} is the boiling point elevation of the brine leaving the heater.

The vapor temperature entering the compressor is higher than the compressor suction temperature by the boiling point elevation

$$T_{C,i} = T_{cb} + (BPE_{sl} - BPE_{cf})/1 \tag{57}$$

where BPE_{sl} amd BPE_{cf} are the boiling point of the brine and slurry.

The power consumption of the compressor if function of the pressure change in the device and the suction temperature as follows [5]

$$\dot{W}_C = \frac{m_v \cdot c_{p,v} \cdot T_{C,s}}{\eta_c} \cdot \left(PR^{\frac{\gamma_v - 1}{\gamma_v}} - 1 \right) \tag{58}$$

where m_v and $c_{p,v}$ are the flow and specific heat of the vapor in the compressor at the suction temperature $T_{C,s}$. PR is the pressure ratio in the compressor and η_c the compressor efficiency assumed as 0.75 [4, 5].

In the separator, the formed salt crystal separates from the slurry forming the recirculation brine

$$\dot{m}_{rb} \cdot C_{rb}/1000 + \dot{m}_s = Slurry \cdot C_{sl}/1000 \tag{59}$$

The recirculation pump transports the recirculating brine to the mixing process with the incoming brine.

$$\dot{W}_{rp} = \frac{\dot{m}_{rb} \cdot \Delta P_{cf}}{\eta_p \cdot \rho_{rb}} \tag{60}$$

The total power consumption of the crystallizer subsystem is the summation of the compressor and pump power consumption.

Cost estimation

The total capital cost for the crystallization system includes the capital cost of the heater, crystallizer, compressor and pump. The purchase cost of the heater is function of the heat transfer area [4]

$$CC_{heater} = 430 \cdot 0.582 \cdot U_{heater} \cdot A_{heater} \tag{61}$$

where U_{heater} is the overall heat transfer coefficient (kW/m^{2}K) and A_{heater} is the heat transfer area. The heat transfer coefficient depends on the temperature of the vapor entering the heater ($T_{C,out}$)[28].

$$U_{heater} = 1.7194 + 3.2063 \cdot 10^{-2} \cdot T_{C,out} - 1.5971 \cdot 10^{-5} \cdot {T_{c,out}}^2 + 1.9918 \cdot 10^{-7} \cdot {T_{C,out}}^3 \tag{62}$$

The following expression allows for estimating the heat transfer area of the evaporator/condenser

$$\frac{(T_{cf} - T_{C,out})}{(T_{cf,h,in} - T_{C,out})} = \exp\left(\frac{-U_{heater} \cdot A_{heater}}{\dot{m}_{cf} \cdot c_{p,cf}}\right) \tag{63}$$

where T_{cf} is is the temperature of the fluid entering the crystallizer and $T_{cf,h,in}$ the temperature of the brine entering the heater. m_{cf} and $c_{p,cf}$ are the mass flow rate and specific heat of the brine.

The purchase cost of the crystallizer is a logarithmic function of the flow entering (m^3/day [2])

$$CC_{crystallizer} = 28531 \cdot \ln \dot{m}_f + 18824 \tag{64}$$

The compressor capital cost is function of the efficiency, pressure ratio and flow in kg/s [4]

$$CC_{compressor} = 7364 \cdot \dot{m}_v \cdot PR \cdot \frac{\eta_C}{1 - \eta_C} \tag{65}$$

The recirculating slurry pump capital cost is function of flow and pressure as follows [29]

$$CC_{cpump} = F_m \cdot F_p \cdot base \tag{66}$$

$$base = 844.31 \cdot Q_p^{0.3726} \tag{67}$$

where Q_p, F_m and F_p are the flow (m^3/h),and material and pressure adjustment factors. For stainless steel material, the material adjustment factor (F_m) is 2.4. The pressure adjustment varies with larger pressures as follows

$$F_p = \begin{cases} 1 & , P_p \leq 10.35 \text{ bar} \\ 0.3968 \cdot P^{0.4132} & , 10.35 \text{ bar} < P_p \leq 300 \text{ bar} \end{cases} \tag{68}$$

The annual operational cost of the BCr subsystem considers power cost, labor, chemicals use, insurance and maintenance as follows [9]

$$OC_{power,BCr} = f_c \cdot energy_{cost} \cdot \dot{W}_{C,BCr} \cdot hours \cdot 365 \cdot \tag{69}$$

$$OC_{labor,BCr} = f_c \cdot 0.01 \cdot m_{d,BCr} \cdot hours \cdot 365 \tag{70}$$

$$OC_{chem,BCr} = f_c \cdot 0.04 \cdot m_{d,BCr} \cdot hours \cdot 365 \tag{71}$$

$$OC_{insur,BCr} = 0.005 \cdot CC_{BCr} \cdot CRF \tag{72}$$

$$OC_{main,BCr} = 0.003 \cdot CC_{BCr} \cdot CRF \tag{73}$$

where f_c is the capacity factor of the system assumed as 0.9, $energy_{cost}$ is the electricity price, $m_{d,BCr}$ the produced freshwater from the BCr subsystem, and CRF the capital recovery factor used for annualizing the capital costs of the subsystem.

S3.2 PV field

S3.2.1 PV area required

The PV area required depends on the PV panel efficiency, irradiation and nominal size (production desired) as follows

$$PV_{area} = \frac{W_{nominal,year}}{\eta_{pvg} \cdot G_{ir}} \tag{74}$$

where $W_{nominal,year}$, η_{pvg} and G_{ir} are the yearly nominal energy production (kWh), the panel efficiency corrected and the incident radiation into the panel. The nominal power depends on the power consumption of the ZLD system, while the incident irradtion considered in this work is the global tilted radiation (kWh/m^2). This geospatial variable indicates the amount of radiation per meter squared received by a PV with optimal inclination based on location [30]. The panel efficiency depends on reference properties and correction factors as follows

$$\eta_{pvg} = \eta_r \cdot (1 - \beta \cdot (T_c - T_{c,ref})) \tag{75}$$

where η_r, β and $T_{c,ref}$ are the reference efficiency, temperature coefficient and cell temperature (S3). T_c is the cell temperature, which is function of the ambient temperature, incident irradiance and the nominal operating cell temperature (NOCT) or overall heat transfer loss provided by manufacturer [31, 32]. This work uses data provided by the System Advisor Model software for a SunPower SPR-X21-335 PV cell [6].

$$T_c = T_{amb} + \frac{NOCT - 20}{800} \cdot G_{ir} \tag{76}$$

Ambient temperature in this work is the average value based on geospatial data.

S3.3 PV cost

The total annualized cost of the PV field is function of the capital cost, operational cost and area investment.

$$TAC_{PV} = CAPEX + OPEX + PV_{area} \cdot land_{cost} \cdot CRF \tag{77}$$

where $land_{cost}$ is the estimated cost of the land [33]. The CAPEX and OPEX for the solar field are 1549 \$/kWp and 14 \$/kWp/year [34, 35].

S3.4 ZLD annual cost estimation and CEPCI correction

The total annualized cost is the summation between the annualized capital cost and the annual operational costs. The annualized capital cost is the summation of the capital cost of every device multiplied by the capital recovery factor (CRF).

The total capital cost of the preconcentration subsystem considers pretreatment system, pumps, pressure exchangers and RO modules as follows

$$
\begin{aligned}
CC_{RO} = & CC_{SWIP} + CC_{HPP,1} + CC_{HPP,2} \\
& + CC_{BP,1} + CC_{BP,2} + CC_{PX,1} + CC_{PX,2} \\
& + N_{units} \cdot (CC_{RO,module,1} + CC_{RO,module,2})
\end{aligned} \tag{78}
$$

where CC_{SWIP} is the capital cost of pretreatment and intake pumping; $CC_{HPP,1}$ and $CC_{HPP,2}$ the capital cost for both high-pressure pumps; $CC_{BP,1}$ and $CC_{BP,2}$ the capital cost for the booster pumps located outside the pressure exchangers; $CC_{PX,1}$ and $CC_{PX,2}$ the capital cost of the pressure exchangers, N_{unit} the number of RO units with 43 vessels and 7 membrane element required for achieving a flow of 2000 m^3/h in every array, and $CC_{RO,module,1}$ and $CC_{RO,module,2}$ the capital cost of every RO module composed by 43 pressure vessels and 7 membrane elements each one. The capital cost correlations are the same as chapter 5. The annualized capital cost for the RO subsystem is

$$ACC_{RO} = CC_{RO} \cdot CRF \tag{79}$$

The annual operational cost of the RO subsystem is the same described in chapter 5.

The total capital cost of the concentration subsystem considers preheaters, evaporator/condenser and compressor as follows

$$CC_{MVC} = CC_{PH} + CC_{C} + CC_{EC} \tag{80}$$

where CC_{PH}, CC_C, and CC_{EC} are the capital costs for the preheaters/regenerators, compressor, and evaporator/condenser. The capital cost correlations are the same as chapter 5. The annualized capital cost for the MVC subsystem is

$$ACC_{MVC} = CC_{MVC} \cdot CRF \tag{81}$$

The annual operational cost of the RO subsystem is the same described in chapter 5.

The total capital cost of the crystallization subsistem considers the heater, crystallizer, compressor and recirculation pump

$$CC_{BCr} = CC_H + CC_{Cr} + CC_C + CC_{rp} \tag{82}$$

where CC_H, CC_{Cr}, CC_C, and CC_{rp} are the capital costs for the heater, crystallizer, compressor, and recirculation pump. The capital cost correlations are avialble in S3.1.4. The annualized capital cost for the MVC subsystem is

$$ACC_{BCr} = CC_{BCr} \cdot CRF \tag{83}$$

The annual operation cost of the BCr subsystem is available in S3.1.4.

The CEPCI correction factor corrects the annualized capital cost to the year 2021. This step is necessary since the correlations are from past years [36]. This work corrects all the annualized capital cost to the year 2021 assuming an origin from year 2000 based on the oldest correlation. The CEPCI values for 2000 and 2021 are 394.1 and 750 [37, 38] .

The CEPCI correction does not apply for the PV system since the CAPEX and OPEX are updated for 2021 [34, 35].

References

[1] Jones, E., Qadir, M., van Vliet, M.T., Smakhtin, V., and Kang, S.m. (2019). The state of desalination and brine production: A global outlook. *Sci. total Environ.* 657:1343–1356.

[2] Chen, Q., Burhan, M., Shahzad, M.W., Ybyraiymkul, D., Akhtar, F.H., Li, Y., and Ng, K.C.

(2021). A zero liquid discharge system integrating multi-effect distillation and evaporative crystallization for desalination brine treatment. *Desalination.* 502:114928.

[3] Chen, Q., Akhtar, F.H., Burhan, M., Kumja, M., and Ng, K.C. (2021). A novel zero-liquid discharge desalination system based on the humidification-dehumidification process: a preliminary study. *Water Res.* 207:117794.

[4] Swaminathan, J., Nayar, K.G., and Lienhard V, J.H. (2016). Mechanical vapor compression—membrane distillation hybrids for reduced specific energy consumption. *Desalination Water Treat.* 57(55):26507–26517.

[5] Thiel, G.P., Tow, E.W., Banchik, L.D., Chung, H.W., and Lienhard V, J.H. (2015). Energy consumption in desalinating produced water from shale oil and gas extraction. *Desalination.* 366:94–112.

[6] National Renewable Energy Laboratory. Golden, C. System advisor model version 2021.12.02 (sam 2021.12.02). Accessed May-24, 2022. https://sam.nrel.gov.

[7] Nafey, A. and Sharaf, M. (2010). Combined solar organic rankine cycle with reverse osmosis desalination process: energy, exergy, and cost evaluations. *Renewable Energy.* 35(11):2571–2580.

[8] Solutions, D.W. (2010). Filmtec™ reverse osmosis membranes. *Technical Manual, Form.* 399(609-00071):1–180.

[9] Vince, F., Marechal, F., Aoustin, E., and Bréant, P. (2008). Multi-objective optimization of ro desalination plants. *Desalination.* 222(1-3):96–118.

[10] Almuhafdah, S., Narayana, G., Ryali, D., and Singh, K. (2017). Performance analysis of ro plant using pressure exchanger as an energy recovery device. *International Journal of Industrial Electronics and Electrical Engineering.*

[11] Malek, A., Hawlader, M., and Ho, J. (1996). Design and economics of ro seawater desalination. *Desalination.* 105(3):245–261.

[12] Lu, Y.y., Hu, Y.d., Xu, D.m., and Wu, L.y. (2006). Optimum design of reverse osmosis seawater desalination system considering membrane cleaning and replacing. *Journal of membrane science.* 282(1-2):7–13.

[13] Lu, Y., Liao, A., and Hu, Y. (2012). The design of reverse osmosis systems with multiple-feed and multiple-product. *Desalination.* 307:42–50.

[14] Poullikkas, A. (2001). Optimization algorithm for reverse osmosis desalination economics. *Desalination.* 133(1):75–81.

[15] Mistry, K.H., McGovern, R.K., Thiel, G.P., Summers, E.K., Zubair, S.M., and Lienhard, J.H. (2011). Entropy generation analysis of desalination technologies. *Entropy.* 13(10):1829–1864.

[16] Pitzer, K.S., Peiper, J.C., and Busey, R. (1984). Thermodynamic properties of aqueous sodium chloride solutions. *J Phys Chem Ref Data.* 13(1):1–102.

[17] Sparrow, B.S. (2003). Empirical equations for the thermodynamic properties of aqueous sodium chloride. *Desalination.* 159(2):161–170.

[18] Nayar, K.G., Sharqawy, M.H., Banchik, L.D., et al. (2016). Thermophysical properties of seawater: A review and new correlations that include pressure dependence. *Desalination.* 390:1–24.

[19] Sharqawy, M.H., Lienhard, J.H., and Zubair, S.M. (2010). Thermophysical properties of seawater: a review of existing correlations and data. *Desalination and water Treatment.* 16(1-3):354–380.

[20] Fabuss, B.M. and Korosi, A. (1966). Boiling point elevations of sea-water and its concentrates. *J. Chem. Eng. Data.* 11(4):606–609.

[21] El-Sayed, Y. (2001). Designing desalination systems for higher productivity. *Desalination.* 134(1-3):129–158.

[22] Jamil, M.A. and Zubair, S.M. (2017). Design and analysis of a forward feed multi-effect mechanical vapor compression desalination system: An exergo-economic approach. *Energy.* 140:1107–1120.

[23] El-Mudir, W., El-Bousiffi, M., and Al-Hengari, S. (2004). Performance evaluation of a small size tvc desalination plant. *Desalination.* 165:269–279.

[24] Jamil, M.A. and Zubair, S.M. (2018). Effect of feed flow arrangement and number of evaporators on the performance of multi-effect mechanical vapor compression desalination systems. *Desalination.* 429:76–87.

[25] Jamil, M.A. and Zubair, S.M. (2017). On thermoeconomic analysis of a single-effect mechanical vapor compression desalination system. *Desalination.* 420:292–307.

[26] Chung, H.W., Nayar, K.G., Swaminathan, J., Chehayeb, K.M., and Lienhard V, J.H. (2017). Thermodynamic analysis of brine management methods: Zero-discharge desalination and salinity-gradient power production. *Desalination.* 404:291–303.

[27] Van der Ham, F. *Eutectic freeze crystallization.* PhD thesis, Delft University of Technology, (1999). http://resolver.tudelft.nl/uuid:5a127123-3d40-41e7-a71b-c91a8fcb1702.

[28] Han, X., Yuan, T., Zhang, D., Dai, Y., Liu, J., and Yan, J. (2021). Waste heat utilization from boiler exhaust gases for zero liquid discharge of desulphurization wastewater in coal-fired power plants: Thermodynamic and economic analysis. *J. Clean. Prod.* 308:127328.

[29] Wan, C.F. and Chung, T.S. (2018). Techno-economic evaluation of various ro+ pro and ro+ fo integrated processes. *Appl. Energy.* 212:1038–1050.

[30] (2022). [Data/information/map] obtained from the "Global Solar Atlas 2.0, a free, web-based application is developed and operated by the company Solargis s.r.o. on behalf of the World Bank Group, utilizing Solargis data, with funding provided by the Energy Sector Management Assistance Program (ESMAP). For additional information: https://globalsolaratlas.info.

[31] Belmili, H., Haddadi, M., Bacha, S., Almi, M.F., and Bendib, B. (2014). Sizing stand-alone photovoltaic–wind hybrid system: Techno-economic analysis and optimization. *Renew. Sust. Energ. Rev.* 30:821–832.

[32] Watson, S., Bian, D., Sahraei, N., Buonassisi, T., Peters, I.M., et al. (2018). Advantages of operation flexibility and load sizing for pv-powered system design. *Sol Energy.* 162:132–139.

[33] Nolte, C. (2020). High-resolution land value maps reveal underestimation of conservation costs in the united states. *Proc. Natl. Acad. Sci. U.S.A.* 117(47):29577–29583.

[34] Suri, M., Betak, J., Rosina, K., Chrkavy, D., Suriova, N., Cebecauer, T., Caltik, M., and Erdelyi, B., (2020). *Global Photovoltaic Power Potential by Country (English)*. Energy Sector Management Assistance Program (ESMAP) Washington, D.C. : World Bank Group.

[35] IRENA. (2021). Renewable power generation costs in 2020. *International Renewable Energy Agency: Masdar City, Abu Dhabi*.

[36] El-Dessouky, H.T. and Ettouney, H.M., (2002). *Fundamentals of salt water desalination*. Elsevier.

[37] Vatavuk, W.M. (2002). Updating the ce plant cost index. *Chem. Eng.* 109(1):62–70.

[38] Chemical Engineering: Essential for the CPI Professional. 2021 CEPCI UPDATES: SEPTEMBER (PRELIM.) AND AUGUST (FINAL). https://www.chemengonline.com/2021-cepci-updates-september-prelim-and-august-final/, (2021). Accessed: May-24-2022.
